# Cellulose-Based Hydrogels for Wastewater Treatment: A Focus on Metal Ions Removal

**DOI:** 10.3390/polym16091292

**Published:** 2024-05-05

**Authors:** Francesca Persano, Cosimino Malitesta, Elisabetta Mazzotta

**Affiliations:** Laboratory of Analytical Chemistry, Department of Biological and Environmental Sciences and Technologies (Di.S.Te.B.A.), University of Salento, via Monteroni, 73100 Lecce, Italy; francesca.persano@unisalento.it (F.P.); cosimino.malitesta@unisalento.it (C.M.)

**Keywords:** cellulose-based hydrogels, heavy metal adsorption, wastewater treatments, adsorption capacity, metal ion removal

## Abstract

The rapid worldwide industrial growth in recent years has made water contamination by heavy metals a problem that requires an immediate solution. Several strategies have been proposed for the decontamination of wastewater in terms of heavy metal ions. Among these, methods utilizing adsorbent materials are preferred due to their cost-effectiveness, simplicity, effectiveness, and scalability for treating large volumes of contaminated water. In this context, heavy metal removal by hydrogels based on naturally occurring polymers is an attractive approach for industrial wastewater remediation as they offer significant advantages, such as an optimal safety profile, good biodegradability, and simple and low-cost procedures for their preparation. Hydrogels have the ability to absorb significant volumes of water, allowing for the effective removal of the dissolved pollutants. Furthermore, they can undergo surface chemical modifications which can further improve their ability to retain different environmental pollutants. This review aims to summarize recent advances in the application of hydrogels in the treatment of heavy metal-contaminated wastewater, particularly focusing on hydrogels based on cellulose and cellulose derivatives. The reported studies highlight how the adsorption properties of these materials can be widely modified, with a wide range of adsorption capacity for different heavy metal ions varying between 2.3 and 2240 mg/g. The possibility of developing new hydrogels with improved sorption performances is also discussed in the review, with the aim of improving their effective application in real scenarios, indicating future directions in the field.

## 1. Introduction

Growing industrial activity together with the reduced availability of drinking water sources have made water pollution a serious problem that requires new effective solutions. The industry is responsible for approximately 20% of global water consumption, continuously releasing various pollutants into water systems, thus resulting in irreparable damage to ecosystems. Contaminants, including heavy metals, are continuously released into the environment through wastewater resulting from human and industrial activities [1]. The presence of heavy metal ions in wastewater inevitably leads to their entry into the surrounding environment and ecological cycles, being subsequently absorbed by animals, microorganisms, and plants.

Heavy metal ions, such those of lead, copper, arsenic, cadmium, and nickel, tend to accumulate in living organisms and, due to their toxicity, high permeability, and lack of degradability, lead to a series of physiological disorders, causing alterations in development, constituting a serious threat to human health [2,3,4,5,6]. In fact, evidence accumulated over the years links heavy metals to various diseases that afflict the human population, such as cancer, anemia, and hypertension, as well as damage to the nervous system and kidney failure. It follows that effective removal of heavy metal ions from wastewater before their release into water bodies would bring benefits to both human health and the environment itself [7,8].

Furthermore, although the earth is covered by 70% water, 97% is salty and only 3% is available for human use. The limited availability of water for human use, along with the large volumes of contaminated water resulting from various industrial activities highlight the need for advanced treatments in the decontamination of wastewater.

Most conventional strategies used for wastewater treatment, such as chemical precipitation, membrane separation, ion exchange, and electrochemical treatments, present limitations which make their scaling up difficult, thus discouraging their application in real contexts in which large water volumes have to be treated, preferably with simple and low-cost procedures based on reusable materials, allowing for reductions in the required time and costs [9,10,11,12,13].

Chemical precipitation, for example, despite being an economical method, requires additional costs for the disposal of the large quantities of sludge produced during the purification process itself [14]. Membrane separation is a pressure treatment method whose efficacy strongly depends on the selection of the membrane, and large-scale application is complicated. This process is based on the principle of selective permeability of membranes, which allows for the selective passage of certain components while hindering or preventing the passage of other components. Some membranes may not be able to selectively separate all types of heavy metals present in water. Ion exchange involves the use of solid surfaces that act as ion exchangers and are characterized by high selectivity toward certain ionic species, meaning that only certain types of ions can be adsorbed [15,16]. This can be a disadvantage as multiple steps or different materials are required to effectively treat a mixture of heavy metals, making the process more complex and expensive. Electrochemical treatments, including electrodeposition, electrocoagulation, electroflotation and electrooxidation, have the advantage of possessing a short process time and a simple operation, with the possibility of fully automation with no additional chemicals required. Nonetheless, the side reaction of hydrogen generation and oxygen generation negatively affecting the efficiency of the process have to be taken into account in electrodeposition, as the high energy consumption affects mainly the electrocoagulation process, while most of the processes suffer from electrode passivation, resulting in a subsequent need of periodic replacement [17,18].

In contrast, adsorption-based strategies represent simple, low-cost, easy to implement, and effective methods in the removal of heavy metal ions, which justify the growing research interest toward this approach during recent years. In the adsorption method, the treated aqueous matrix is simply stirred in the presence of an adsorbent able to bind to contaminants due to the presence of surface functional groups acting as active sites, until the saturation of the binding sites is reached [19]. To achieve an efficient and convenient adsorption process, desired adsorbent properties are a large surface area, numerous active sites, biodegradability, and easy assembly and availability. Traditional adsorbents, such as artificial resin, activated carbon, zeolite, diatomaceous earth, alumina, and silica gel, although widely used in the last few years, are affected by reduced adsorption capacity and poor regeneration performance [20,21].

In this context, porous adsorbents with a three-dimensional structure and large surface area enabling the exposure of numerous active sites are recently emerging, due to their greater adsorption capacity and reduced adsorption time achieved by rapid diffusion kinetics throughout the adsorbent. The efforts of increasing the number of surface functional groups and controlling the surface area for improving the adsorption process have led to the design of tailored porous materials such as metal organic frameworks (MOFs) or covalent organic frameworks (COFs), although their application in water treatment still requires further studies to increase their stability and decrease production costs, along with a conducting critical evaluation of their toxicity.

Among porous adsorbent materials, hydrogels occupy a key role, combining a high adsorption capacity with reusability, biodegradability, and non-toxicity, reducing environmental impact and promoting sustainable practices in water treatment. Hydrogels are soft, elastic materials made of three-dimensional polymer networks with the extraordinary ability to store large amounts of water (up to hundreds of times with respect to their dry weight) thanks to their hydrophilic polymer structure, all features explaining their potential and their increasing applications as adsorbents for water remediation [22,23]. Among the wide panorama of polymers used for hydrogel development, cellulose—a well-known polysaccharide made up of glucose units held together by ß-1,4-glucosidic bonds—occupy a prominent role [24,25], being the most abundant natural polymer on earth (the main component of plant cell walls) with high biocompatibility, biodegradability, low cost, and controllable properties. Related to this, cellulose, its derivatives, and nanocellulose-based hydrogels have been conveniently exploited in a plethora of uses ranging from the biomedical field (administration of therapeutic agents, in tissue engineering, implant materials, and wound dressings) to energy and environmental applications [26,27,28]. Specifically, the presence of numerous surface functional groups capable of coordinating metal ions in water, together with the possibility of easily obtaining an interconnected porous structure, has promoted the application of cellulose and its derivatives in the production of hydrogels for water decontamination in terms of heavy metals [29,30].

The present review focuses on the main developments in the field, presenting first the main manufacturing methods of cellulose-based hydrogels, then describing the mechanisms involved in heavy metal adsorption processes, and highlighting some recent selected applications. Prospective future trends in the field are also discussed, along with indications of possible directions to take to bridge the gap between laboratory research and practical application and fully exploit the effectiveness of cellulose-based hydrogels and their derivatives in the removal of metal ions from water.

## 2. Synthesis of Cellulose- and Cellulose Derivative-Based Hydrogels

### 2.1. Structure of Cellulose and Its Derivatives

**Figure 1:**
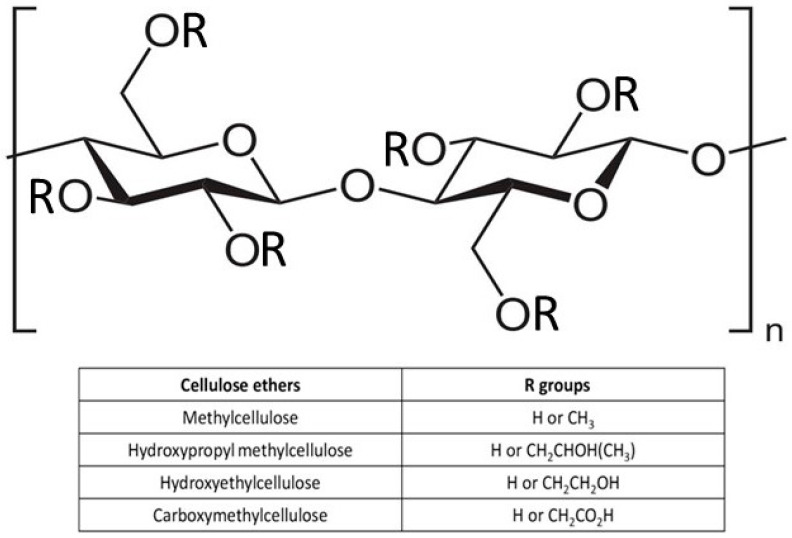
General chemical structure of ether cellulose. Functional groups (R) of the main cellulose ethers are reported.

Cellulose hydrogels can be developed from a pure cellulose solution through physical cross-linking thanks to the presence of a many hydroxyl groups (–OH) that allow the polymer network to be connected by hydrogen bonding. The –OH groups can be functionalized with various chemical modifications [31,32,33,34,35,36,37,38,39], often consisting of partial or total etherification, through reactions with organic species, such as ethyl and methyl units [40]. Cellulose ethers, based on their substituents, can be classified as single ethers or mixed ethers. Single cellulose ethers involve the substitution of a single functional group in the cellulose unit, while mixed ethers involve the substitution of more than one functional group. Individual ethers include alkyl ethers such as methyl cellulose (MC) and hydroxyalkyl ethers such as hydroxypropyl cellulose, while mixed ethers are mainly represented by carboxymethylcellulose (CMC), hydroxyethyl cellulose (HEC), and hydroxypropyl methyl cellulose [41,42] (Figure 1).

Cellulose ether can be soluble in water depending on the chemical structure of the substituent as well as the type and degree of substitution. Typically, water-soluble cellulose ethers are characterized by a degree of substitution between 0.4 and 2 [43]. MC is the simplest alkyl ether, soluble in various organic solvents and in water depending on the degree of substitution. Specifically, for a degree of substitution between 1.4 and 2.0, MC is soluble in water [44,45,46]. Interestingly, MC has thermo-gelling properties which can be effectively exploited in promoting temperature-controlled hydrogel behavior [47]. CMC is characterized by hydrophilicity, stability in water, elevated chemical stability, biocompatibility, and biodegradability. However, CMC is not soluble in some organic solvents such as ethanol [48]. HEC turns out to be soluble in water and various organic solvents. In addition, HEC can be blended with cellulose, allowing cellulose to be processed without the use of solvents [49].

One fascinating feature of cellulose-based hydrogels is their biodegradability, being degradable by fungi and bacteria present in water, air, and soil, contrarily to acrylate-based hydrogels, typically used at industrial level, which show slow degradation, are non-biodegradable, and are therefore considered potential pollutants of water and soil. Storing cellulose in soil for 10 weeks leads to its degradation by 82–95%, depending on the type of soil [50,51]. When applied in decontamination processes, such a feature represents an additional benefit allowing us to reduce the environmental impact of residual materials and thus improving the environmental sustainability of the whole process.

The approaches for preparing cellulose-based hydrogels can be essentially divided into two types, namely, physical and chemical cross-linking methods [30,52].

### 2.2. Physically Cross-Linked Hydrogels

Physically cross-linked hydrogels are reversible hydrogels, assembled through the interweaving of polymer chains by means of intra- and intermolecular hydrogen bonding, ionic bonding, hydrophobic interaction, and non-permanent forces, such as electrostatic and gravitational forces [53,54]. They are synthesized without the use of chemical cross-linking agents, which, being toxic in most cases, should be completely removed from the system before use. Therefore, the resulting hydrogels possess increased biocompatibility and reduced or even no toxicity [55,56].

**Figure 2:**
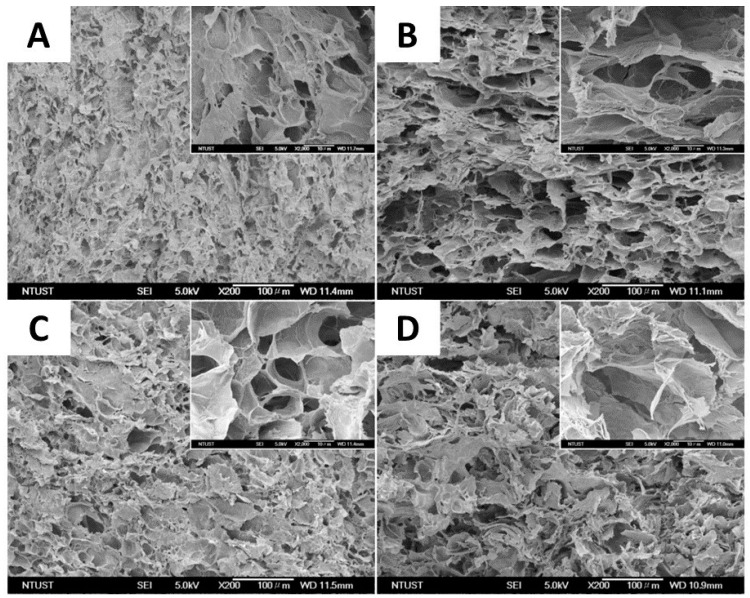
SEM images of the cross-section of the different PVA/CMC composite formulations: (**A**) PVA (1:0, PVA/CMC), (**B**) P2C1 (2:1, PVA/CMC), (**C**) P1C1 (1:1, PVA/CMC), and (**D**) P1C2 (1:2, PVA/CMC) (scale bar: 100 μm), from ref. [57].

One of the most widely used methods of physical cross-linking for the production of cellulose-based hydrogels with elevated absorbency is the cryogenic method (repeated freeze–thaw cycles) [57]. This process leads to the formation of hydrogels due to phase separation in the polymer solution after repeated freezing and thawing cycles. Thus, the formation of polymer crystallites occurs when polymer solutions are exposed to low temperatures, creating insoluble polymer networks. Furthermore, freeze–thaw cycles allow a porous structure to be created in hydrogels thanks to the space left by ice crystals that melt during the thawing phase. In a study conducted by Guan et al. [58], a hemicellulose-based hydrogel with remarkable mechanical properties was developed through repeated freeze/thaw cycles, which led to the formation of a system with a compact structure characterized by high thermal stability and mechanical resistance. The maximum compressive strength was reached after nine freeze/thaw cycles, highlighting the packing of the physically cross-linked polymer chains induced by repeated freeze/thaw cycles. The group of Wang et al. applied the cryogenic approach to the synthesis of a composite hydrogel consisting of poly(vinyl alcohol) (PVA) and carboxy methyl cellulose (CMC) (Figure 2). Specifically, a mixed aqueous solution of PVA and CMC was frozen at −20 °C for 6 h and thawed for 1 h at room temperature, for five freeze/thaw cycles [57]. Freezing the polymer solutions caused crystals to form, generating insoluble hydrogels. The physical properties and degree of swelling of the gels were clearly influenced by the proportions of PVA and CMC. Pure PVA hydrogels showed a swelling degree of 416%, while those with 1:2 PVA/CMC ratio reached 1437%. The results indicate that an increase in CMC content led to a reduction in gel fraction and an increase in the degree of swelling for PVA/CMC hydrogels with different CMC proportions. This is due to the increasing hydrophilicity of CMC and a lower cross-linking density of the hydrogel as the amount of PVA decreases. Cross-section SEM images of the prepared hydrogels highlight that all PVA/CMC hydrogels exhibited a spongy structure with high porosity (Figure 2).

The main advantages of hydrogels produced through physical cross-linking over chemically cross-linked ones include ease of fabrication, biodegradability, biocompatibility, reduced toxicity, post-process mass modification, and remodeling. In contrast, in physical cross-linking processes, weak interactions between polymer chains, such as hydrogen bonds or physical interactions, allow the hydrogel structure to be formed more easily. Furthermore, physical cross-linking makes hydrogels more biodegradable, as the weak interactions within the polymer network are more susceptible to biodegradation compared to covalent bonds [59]. On the other hand, physically cross-linked hydrogels usually suffer from fast dissolution and reduced stability due to the lack of chemical bonding within the hydrogel network [60].

However, in a recent work, the group of Zhang et al. [61] proposed the synthesis of a physically cross-linked CMC-Fe^3+^/polyacrylamide dual-network hydrogel via a two-step process. In particular, Fe^3+^ cross-linked CMC forms the first network, while the hydrophobic polyacrylamide forms the second network to preserve the integrity of the resulting hydrogel. This system showed excellent mechanical properties. The mechanical properties were evaluated by tensile and compressive tests. Compared with the physically cross-linked single CMC/polyacrylamide hydrogel, the elastic modulus and toughness of the CMC-Fe^3+^/polyacrylamide hydrogel increased by 420% and 94%, respectively.

### 2.3. Chemically Cross-Linked Hydrogels

Chemical cross-linking involves the formation of covalent bonds between the crosslinking agents and the polymer chains which impart improved stability and great mechanical strength to the resulting hydrogels [62]. Chemically cross-linked systems do not melt or disintegrate if treated with heat, and do not dissolve in water without first breaking the covalent bonds. For this reason, they are called perpetual systems [63]. Chemical cross-linking can occur through esterification, radical polymerization, Michael addition, etc. In the case of cellulose and its derivatives, commonly used cross-linking agents are citric acid, succinic acid, epichlorohydrin, polyethylene glycol diacrylate, divinyl sulfone, and glutaraldehyde (EGDE) [30,64].

**Figure 3:**
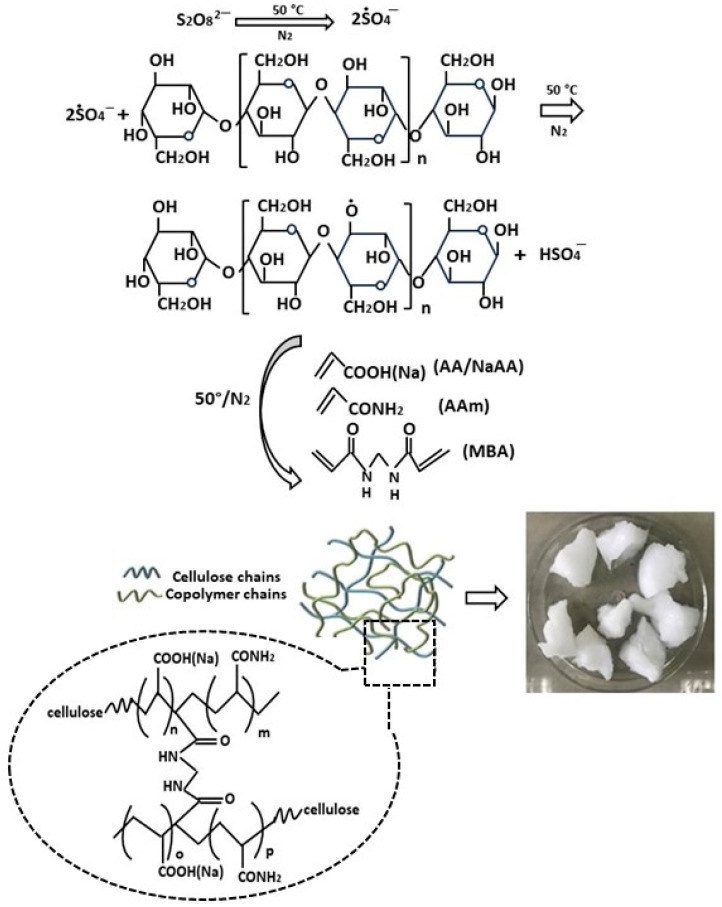
A schematic of the synthesis reaction of bioadsorbents from the modified cellulose through simultaneous cross-linking and grafting of both acrylamide and acrylic acid, from ref. [65]. Aam: acrylamide; AA: acrylic acid; MBA: N,N′-methylene bisacrylamide.

The group of Zhao et al. prepared a modified cellulose-based hydrogel through an innovative method of simultaneous cross-linking and grafting of acrylamide and acrylic acid (Figure 3) [65]. The synthesis was carried out by adding ammonium persulfate to an aqueous solution of microcrystalline cellulose to generate hydroxyl radicals under magnetic stirring at 50 °C. Subsequently, a mixed solution of acrylic acid (AA), acrylamide (AM), and N,N′-methylene bisacrylamide (MBA) was slowly added, continuing stirring at 50 °C for 2 h until complete polymerization. The physicochemical characterization of the hydrogels successfully confirmed the efficient grafting of acrylate and acrylamide monomers onto the initial cellulosic material. Scanning electron microscopy (SEM) analysis revealed a rough and irregular porous structure. The thermal characteristics of cellulose-based hydrogels were examined through thermogravimetric analysis (TGA). While the unmodified hydrogel showed a high decomposition rate in the temperature range from 240 °C to 360 °C, for the modified hydrogel, the main decomposition stage was significantly prolonged and the rate was reduced. The good thermal stability observed represents a positive element for practical applicability, since it gives them greater resistance to temperature variations.

**Figure 4:**
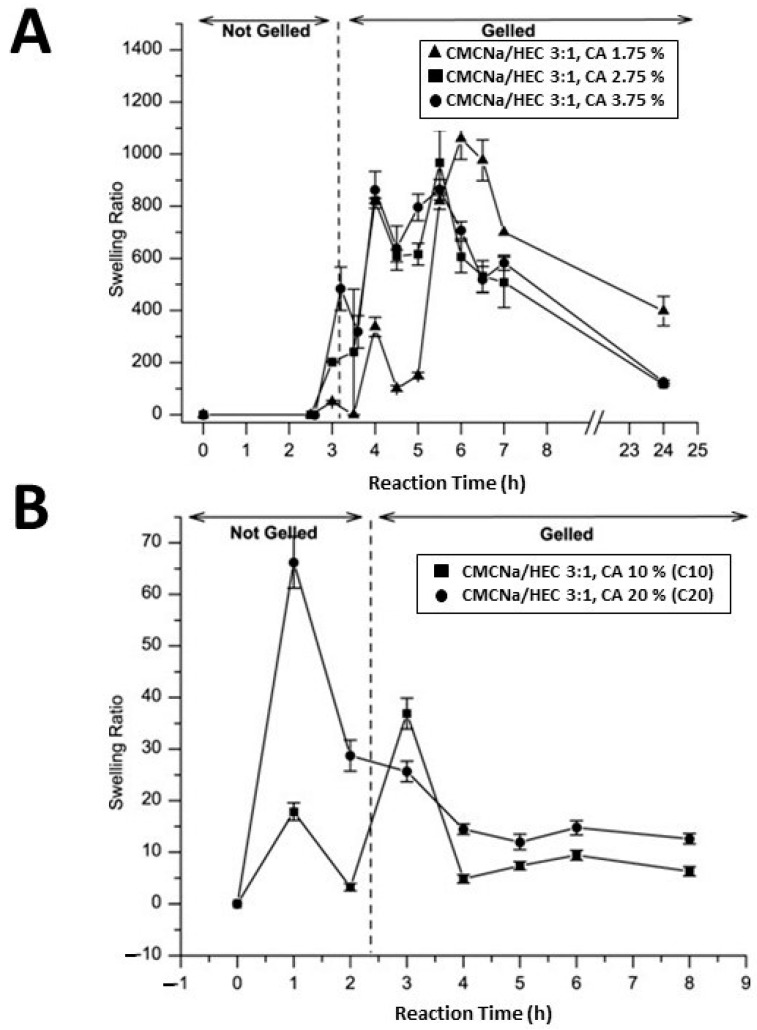
Swelling ratio versus reaction time of CMC/HEC (weight ratio 3:1) hydrogels with 1.75%, 2.75%, and 3.75% (*w*/*w*) of CA cross-linker concentration (**A**). Swelling ratio versus reaction time of CMC/HEC (weight ratio 3:1) hydrogels with 10% and 20% (*w*/*w*) of CA cross-linker concentration (**B**), from ref. [68].

Citric acid (CA), widely used in the food and pharmaceutical sectors, is a highly efficient cross-linking agent. Present in nature, such as in lemon juice where it constitutes approximately 5% of the composition, CA is produced on a commercial scale through the fungal fermentation of glucose. Numerous studies have employed CA as a cross-linking agent in various cellulose-based hydrogels as, compared to other cross-linking agents, CA offers the advantage of being inexpensive and non-toxic [66,67,68,69]. For example, Coma et al. [66] conducted cross-linking of hydroxypropyl methylcellulose with citric acid (CA) in order to reduce the water sensitivity of packaging materials based on water-soluble cellulose derivatives and, above all, to avoid partial solubilization of films in food products. This was achieved by attaching the carboxylic acid moiety to the hydroxypropyl methylcellulose polymer via an esterification reaction of the first cyclic anhydride. Such a reaction exposes a new carboxylic acid unit in the CA, which has the chemical connectivity necessary to form a new intramolecular anhydride moiety with the adjacent carboxylic acid unit. Subsequently, further reaction with cellulose ether led to cross-linking. This process resulted in limiting the affinity of the films with water, since the –OH groups of hydroxypropyl methyl cellulose are involved in the ester bonds. The study demonstrated that cross-linking offers significant benefits in reducing the water solubility of ether cellulose films while simultaneously improving moisture barrier properties by approximately 34%. Demitri et al. [68] synthesized a biodegradable HEC/CMC hydrogel using CA as a cross-linking agent. Their results highlighted that the concentration of CA and the reaction time influence the degree of swelling of cellulose-based hydrogels. In particular, the hydrogels showed a high degree of swelling at low concentrations of CA (1.75%, 2.75%, and 3.75% by weight polymer); a swelling degree of 900 was achieved in the presence of 3.75% CA (Figure 4A), while CA concentrations lower than 1.75% resulted in weak cross-linking associated with insufficient mechanical properties. On the other hand, higher CA concentrations (10% and 20% by weight polymer) produced hydrogels with a low degree of swelling (Figure 4B), indicating high cross-linking. It was also observed that this type of hydrogel, after swelling, had good rigidity and retained the original shape of the tub of synthesis. Subsequently, CA as a cross-linking agent was successfully used in another study for the synthesis of CMC and HEC hydrogels. In particular, chemical treatment with CA induced –COOH functional groups, with an increase in hydrophilicity and surface roughness of the resulting materials [69], as revealed by contact angle and Atomic Force Microscopy (AFM) measurements. The results indicated a significant decrease in contact angle, approximately 50% and 67%, after cross-linking between HEC/HEC-CA and CMC/CMC-CA hydrogels, respectively. Furthermore, AFM profiles evidenced that the addition of CA resulted in an increase in roughness, measured by root mean square (RMS), with average values of 31.82, 39.72, 9.98, and 16.56 nm for CMC, CMC-CA, HEC, and HEC-CA, respectively.

Yan et al. produced a hydrogel based on hydroxypropyl cellulose cross-linked with epichlorohydrin and ammonia in an aqueous sodium hydroxide solution [70]. A temperature-induced phase separation (TIPS) process was applied to synthesize a microporous cross-linked hydrogel which involves the creation of a gel-like structure through the phase transition of the polymer. Typically, the process starts with a solution containing hydroxypropyl cellulose in water or a compatible solvent. Next, the system is heated or cooled to a specific temperature, often above or below the critical solution point of the polymer. This temperature change induces phase separation, leading to the formation of a three-dimensional network of interconnected polymer chains which trap water or the solvent within the gelatinous structure.

Epichlorohydrin as a cross-linking agent was also used for the synthesis of a new superabsorbent system based on native cellulose and quaternized cellulose (cellulose that has been chemically modified by adding quaternary ammonium groups to its structure) in a NaOH/urea aqueous solution to overcome the limitations of reduced biocompatibility, poor mechanical properties, and a lack of antimicrobial activity associated with the superabsorbent hydrogel normally used in the production of disposable diapers [71]. Increasing the density of the cross-linking network using epichlorohydrin led to an improvement in the mechanical properties of the hydrogel. In addition, the introduction of quaternized cellulose within the hydrogel networks not only improved the swelling ratio due to the electrostatic repulsion of the quaternary ammonium groups, but also endowed the hydrogel with antimicrobial properties. The presence of antimicrobial activity was due to the attraction of the anionic moieties of the microbial membranes toward the internal pores of the polycationic hydrogel, which caused its rupture.

**Figure 5:**
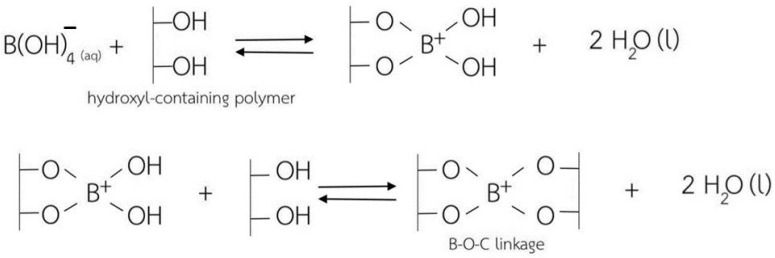
A schematic representation of the cross-linking mechanism between the hydroxyl groups of the cellulose chains and borax as a cross-linking agent, from ref. [72].

In this regard, cellulose-based hydrogels with interesting antibacterial properties (for Gram-positive bacteria) were developed in a very recent study, in which borax (sodium tetraborate) was used as a cross-linking agent [72]. Borax is commonly used as a non-toxic food additive, being cheap and soluble in water. Cellulose-based hydrogels were synthesized using cellulose extracted from water hyacinth via a dissolution process in the presence of urea and sodium hydroxide, with borax employed to generate cross-linking between the –OH groups of the cellulose molecules (Figure 5). The use of borax gave the cellulose-based hydrogel products extraordinary superabsorbent properties, with a swelling ratio of 325% for the non-cross-linked hydrogels and a swelling ratio of approximately 900% for the cross-linked ones.

## 3. Hydrogels Based on Cellulose and Cellulose Derivatives for Water Decontamination of Heavy Metals

In recent years, gels based on cellulose and its derivatives have increasingly attracted research interest for their possible application as heavy metal absorbents in wastewater decontamination processes, thanks to several advantageous characteristics such as a porous structure, numerous functional groups, and high absorption capacity [73,74,75,76,77,78,79,80,81].

Hydrogels, similar to ion exchange resins, can subtract contaminants from water bodies thanks to the presence of functional groups, including carboxyl, hydroxyl, and amino groups [82,83,84,85,86,87,88], which can establish different interactions with metal ions [89]. In addition, through the modification of the functional groups, the properties of the hydrogel can be suitably modified to further improve its interaction ability with metal ions, for example introducing chelating functional groups or opportunely charged moieties able to bind to metal ions through electrostatic interaction. Furthermore, the unique three-dimensional structure of the hydrogel allows for a uniform distribution of metal ions throughout the system, preventing aggregation and oxidation [62]. Indeed, metal ions uniformly distributed within the hydrogel matrix are less susceptible to interaction with oxygen or other oxidizing chemical species present in the surrounding environment, helping to prevent their oxidation. If metal ions are oxidized during the removal process, they may lose their ability to be effectively captured by adsorbents, thus reducing the overall treatment efficiency. Also, the water present in the hydrogel plays a role in this sense, not only providing support to the hydrogel itself, allowing it to maintain a certain shape, but also ensuring the presence of nanotransport channels for the free diffusion of metal ions and participating in the formation of hydrogen bonds, thus constituting additional active sites for the interaction with the target ionic species [90].

In cellulose-based hydrogels, electrostatic attraction usually occurs between unprotonated carboxyl groups (–COO^−^) and metal cations [79,80]. Other mechanisms, such as complexation and microprecipitation, have been suggested for the absorptive removal of heavy metals by using cellulose-based hydrogels. Complexation can occur between the lone pair of O electrons and metal ions. Under some circumstances, for example when the temperature, pH, or concentration of other substances vary, the stability of the metal complex may decrease, leading to metal recrystallization as solid particles with the formation of small solid particles near the metal complexation sites (microprecipitation) [81,91].

In the next sections, brief descriptions will be provided on the adsorption kinetics and isotherms that govern the adsorption processes of heavy metal ions by hydrogels based on cellulose and its derivatives. Finally, a broad overview of the application of such materials as adsorbents for heavy metal ions, both in non-composite and composite systems, will be presented.

### 3.1. Kinetics and Adsorption Isotherms

The study of adsorption kinetics analyzes the variation in the distribution of metal concentration between the adsorbate–adsorbent phases over time, keeping temperature and pressure constant. This study is essential to determine the adsorption rate (and consequently the efficiency) and understand the adsorption mechanism. It provides valuable information on possible adsorption mechanisms and rate-limiting steps during the process. Furthermore, it helps to select the optimal process parameters and conditions for large-scale removal.

The mathematical models, their meaning, and their application in cellulose-based adsorbents and its derivatives are briefly summarized in Table 1.

The adsorption isotherm is fundamental to describe the interaction of solutes with adsorbents and to optimize their use. Isotherm data are typically correlated using a variety of models, with the most suitable model able to examine the adsorption behavior. These isotherms provide qualitative information on the adsorption capacity of adsorbents and allow for the study of equilibrium adsorption data. Table 2 presents equations for various mathematical models, along with their significance and examples of adsorption isotherm studies focusing on the adsorption of heavy metals by cellulose-based hydrogel and its derivatives.

### 3.2. Non-Composite Hydrogels

**Figure 6:**
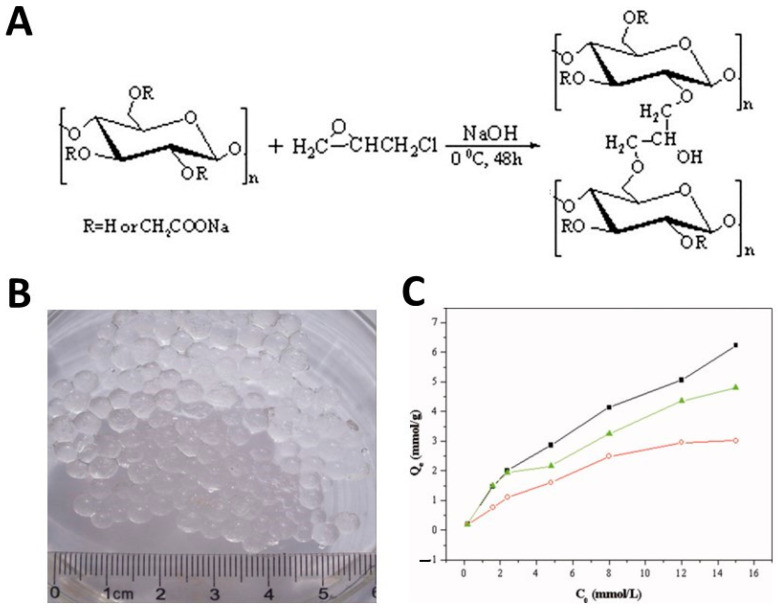
A schematic diagram of the preparation of CMC hydrogels cross-linked with epichlorohydrin (ECH) (**A**). A photograph of CMC-based hydrogel spheres (**B**). The impact of the initial concentration on metal ion adsorption capacity of CMC hydrogel beads: (▪) Cu^2+^, (▴) Pb^2+^, and (○) Ni^2+^ (**C**), from ref. [96]. The quantity of metal ions adsorbed at adsorption equilibrium is reported in ordinates.

A cellulose derivative commonly used in the development of hydrogels for the purification of wastewater from cationic heavy metal ions is CMC due to its solubility in water and the presence on the surface of a large number of carboxymethyl groups. Environmentally friendly CMC-based hydrogels were produced by the group of Yang et al. [96] through a strategy of reverse cross-linking in suspension using epichlorohydrin as cross-linking agent (Figure 6A). CMC solutions were prepared by directly mixing NaOH and CMC with deionized water. Then, epichlorohydrin was added to the CMC solution under stirring and the mixture was dispersed in a continuous fluid wax phase. The resulting hydrogel was in spherical form, with a diameter of about 4 mm (Figure 6B). FTIR spectra confirmed the formation of a new ether bond in the reaction between epichlorohydrin and CMC, while the analysis of X-ray diffraction (XRD) spectra revealed that the adsorption of cationic metal ions on the oxygen atom of the –COOH group upon coordination resulted in changes in the crystallinity patterns of the hydrogels. Furthermore, the authors demonstrated that the adsorption of heavy metals strongly depends on the pH of the solution. As expected, by increasing the pH, the deprotonation of the –CH_2_–COOH groups led to an improvement in the adsorption of positively charged heavy metal ions due to electrostatic attraction. The adsorption capacity increased also with the increase in the initial concentration of the heavy metal ions (Figure 6C). The maximum amount of adsorption reported for CMC spheres for heavy metal ions was 6.49, 5.15, and 4.06 mmol/g for Cu^2+^, Pb^2+^, and Ni^2+^, respectively.

Sphere-shaped hydrogels were also synthesized by another research group [99] who proposed functionalized titanate–cellulose hydrogel microspheres to treat oily wastewater containing heavy metals. Hydrogel microspheres were prepared by using a sol–gel process using a dissolved ionic liquid cellulose solution as the base material. Specifically, bamboo fibers were dissolved in 1-butyl-3 methylimidazolium chloride at 85 °C through magnetic stirring, and the obtained cellulose solution was added to the titanate nanotubes. Finally, the mixture was added dropwise to a regeneration bath filled with deionized water. The functionalized microspheres were tested for treating oily wastewater containing various types and amounts of metal ions. The results demonstrated that the functionalized microspheres had a maximum Cu^2+^ ion adsorption capacity of 176.4 mg/g, higher than that of pure titanate nanotubes, with a faster adsorption behavior, removing 98% of Cu^2+^ ions within 20 min, compared to the 30 min taken by the pure titanate nanotubes. The maximum adsorption capacities of the microspheres for Pb^2+^, Sr^2+^, Fe^2+^, Cd^2+^, Zn^2+^, and Cr^3+^ ions were 613.5, 131.3, 158.2, 294.6, 187.8, and 97.8 mg/g, all higher than those of pure nanotubes, for which they were 462.3, 94.2, 124.5, 231.8, 128.7, and 64.4 mg/g, respectively. Gelation of the titanate adsorbent by cellulose significantly improved the adsorption capacity of the nanotubes, giving the functionalized microspheres superior performance in terms of adsorption of a variety of metal ions. The excellent adsorption properties of the functionalized microspheres were attributed to the presence of numerous interconnected porous channels and to an ion exchange process involving the replacement of Na^+^ or H^+^ ions in the intermediate layers of the titanate nanotubes [100] with metal cations. The microspheres showed excellent recovery, maintaining a Cu^2+^ ion removal capacity of 70% after seven cycles. For their regeneration, the microspheres were equilibrated with HCl (pH = 1) and NaOH (pH = 13) sequentially for 5 h, then washed three times with deionized water.

Recently, a CMC-based bead-like adsorbent has been produced using Al(III) as a cross-linker [95]. The cross-linking reaction with Al(III) occurs through the complexation between the aluminum ions and the carboxyl groups of CMC. These coordinative bonds act as bridges between polymer chains, increasing the strength and stability of the entire hydrogel structure. In particular, the system was tested in adsorption studies of Pb^2+^, Ni^2+^, and Co^2+^ ions from aqueous solutions. To obtain regular CMC spheres, an aqueous solution of CMC at the desired concentration was injected into a 3% (*w*/*v*) aluminum nitrate solution through a peristaltic pump, under constant stirring. The results obtained demonstrated a maximum adsorption capacity of 550 mg/g for Pb^2+^, 620 mg/g for Ni^2+^, and 760 mg/g for Co^2+^. Furthermore, for the three metals, adsorption increased as the initial concentration and pH increased. This adsorbent also showed extraordinary reusability after four adsorption–desorption cycles upon CMC sphere regeneration with a 0.1 M EDTA solution, with only a slight reduction in adsorption capacity for the three heavy metal ions.

The group of Hara et al. [101] evaluated the absorption capacity of Cu^2+^ ions on a CMC gel cross-linked through irradiation with γ-rays without the use of toxic cross-linkers. A certain amount of CMC was dissolved in water, producing aqueous solutions of CMC at different concentrations (between 1% and 20% by weight). Irradiation with γ-rays (∼20 kGy) did not result in a significant change for CMC concentrations between 1% and 6 wt%, while for concentrations between 10% and 20% by weight, the formation of lumps within the irradiated solution was observed. Therefore, the study of heavy metal capture was conducted using samples prepared with a CMC concentration of 10–20% by weight. The amount of captured Cu^2+^ ions was higher with increasing concentrations of CMC and with increasing doses of γ-rays. These results suggested the involvement of –COOH groups in the Cu^2+^ ion adsorption mechanism. According to the authors, two different models of capture of the Cu^2+^ ion can be considered. The first model involves each single Cu^2+^ ion being adsorbed by a –COOH group, so as the number of –COOH groups increases, the amount of captured Cu^2+^ ions increases, regardless of the degree of cross-linking of the CMC gel. The second model involves two or more –COOH groups being engaged in the adsorption of a single Cu^2+^ ion with a chelation mechanism. Also in this case, the amount of captured Cu^2+^ ions increases with the number of –COOH groups; however, since two or more –COOH groups are involved in the interaction with Cu^2+^ ions, it is also possible to observe a dependence of the number of adsorbed ions on the degree of CMC cross-linking. Moreover, when using a higher dose of γ-rays for irradiation, greater cross-linking is achieved, conferring a more tight and rigid structure to the polymer network, on which the chelation of Cu^2+^ ions occurs more efficiently and stably than on less cross-linked CMC. In other words, increased cross-linking leads to a more rigid structure of CMC polymers, stabilizing their arrangement and allowing for more effective chelation of Cu^2+^ ions.

The group of Kamel et al. produced cyanoethyl cellulose with two different degrees of substitution (0.37 and 0.60) starting from cotton linters [102]. The ionic xanthate method has been exploited to graft acrylonitrile onto cyanoethyl cellulose. Briefly, ion xanthate graft polymerization was performed with a two-step method involving the formation of a cyanoethyl cellulose xanthate solution from the reaction of cyanoethyl cellulose with CS_2_ (carbon disulfide) and NaOH. Subsequently, in the polymerization phase, the solution produced in the first phase was polymerized with acrylonitrile at 30 °C for 2 h with stirring. The impact of grafting conditions was evaluated, such as grafting time, sodium hydroxide concentration, monomer concentration, and temperature. Furthermore, the application of hydrolyzed cyanoethyl cellulose and acrylonitrile-grafted cyanoethyl cellulose on the capture of Cu^2+^ ions from an aqueous solution was carried out. Their results clearly revealed that the amount of Cu^2+^ ions captured by the aqueous solution increased with the increasing degree of substitution of cyanoethyl cellulose, and in addition, acrylonitrile-grafted cyanoethyl cellulose gave significantly higher adsorption than non-grafted cyanoethyl cellulose.

In 2020, a research team extracted cellulose fiber (Cell) from pineapple leaves through an alkaline process and modified it with different groups such as carboxymethyl group (CM) and ethylenediaminetetraacetic acid (EDTA) for the synthesis of Cell-CM and Cell-EDTA, respectively, to be used as heavy metal ion adsorbent materials [93]. An aqueous solution of lead ions (Pb^2+^) and cadmium ions (Cd^2+^) in a single or binary system was used as model wastewater to evaluate their actual adsorption efficiency. For the two forms of modified cellulose, the recorded adsorption efficiency was significantly higher than that of unmodified cellulose. The maximum adsorption capacity of the unmodified extracted cellulose for Pb^2+^ and Cd^2+^ was 5.6 and 4.1 mg/g, respectively. While for Pb^2+^ and Cd^2+^, the maximum absorbed amount was 63.4 and 23.0 mg/g for Cell-CM, and 41.2 and 33.2 mg/g for Cell-EDTA. Furthermore, it was observed that for both metal ions tested, the time required to reach adsorption equilibrium was longer for Cell-EDTA. This is closely related to the adsorption process in the Cell-EDTA system, based on a coordination mechanism which is naturally slower than the ionic bonding occurring in the Cell-CM system. Furthermore, measuring the adsorption in a binary system of Pb^2+^ and Cd^2+^ at different ratios revealed that for the two formulations, the adsorption of Pb^2+^ was higher than that of the Cd^2+^ ion. Finally, the study of the desorption of Pb^2+^ and Cd^2+^ with 1 M HCl revealed that the regenerability of Cell-EDTA was better than that of Cell-CM.

Wang et al. [103] proposed a physical cross-linking strategy for the development of highly robust aerogels (a gel in which the liquid phase is replaced by a gas such that its network remains solid, with only slight or no shrinkage of the gel) with 3D structures for use as an adsorbent material for wastewater decontamination. They combined the action of CA and hydrochloric acid (HCl) for the conversion of cellulose –OH groups into –COOH groups, using high-aspect-ratio cellulose nanofibers obtained from ginger fiber. The combined use of CA and HCl offers the possibility to modulate the properties of the aerogel, such as porosity, swelling ratio, and stability, thus allowing the hydrogel to be adapted to different applications. Carboxylated cellulose nanofiber (CCNF) with the highest aspect ratio of 144 was obtained with a single-step acid hydrolysis technique. With their method, they developed 9-CCNF, 7-CCNF, and 5-CCNF with different carboxyl contents in relation to the different ratios of CA/HCl used (*v*/*v* = 9/1, 7/3, and 5/5). 7-CCNF showed the highest carboxyl content (1.18 ± 0.1 mmol/g) and the highest negative zeta potential (−36 ± 3 mV). The as-obtained carboxylated CNF was then assembled into a stable aerogel with tunable density through hydrogen bonding. The 7-CCNF aerogels also exhibited the best compressive mechanical performance (99.5 kPa at 80% strain) and good shape recovery. In addition, the 7-CCNF aerogel showed a high adsorption capacity for Cu^2+^ (tested as a model of heavy metal cation ion) up to 45.053 mg/g in simulated waters, due to charge neutralization mechanisms and a 3D network trapping effect within mesopores and macropores.

Sharma et al. [104] synthesized nitro-oxidized carboxycellulose nanofibers (NOCNFs) extracted from untreated jute with a nitroxidation method and proved their efficacy for the removal of Pb^2+^ from water. Carboxyl cellulose nanofibers were extracted from untreated jute through a net oxidation process based on sodium nitrite and nitric acid. The resulting nitrous ions can attack the primary OH group of cellulose in C6 with the formation of aldehyde groups (intermediates) and –COOH groups to obtain a mixture of NOCNF. The resulting NOCNF exhibited low crystallinity (35%), high carboxyl content (1.15 mmol/g), and negative surface charge (−70 mV). A suspension of NOCNF (0.23 wt%) was able to rapidly adsorb Pb^2+^ ions in a time interval lower than 5 min from 50 to 5000 ppm, with a maximum adsorption capacity of 2270 mg/g.

With the aim of increasing the number of functional groups able to interact with metal ions in cellulose-based hydrogels, some authors developed thiol-functionalized cellulose sponges by using a room-temperature reaction between TEMPO-oxidized cellulose nanofibrils (TOCNs) and (3-mercaptopropyl)-trimethoxysilane (MPTMS). The method used for obtaining TOCNs was based on the oxidation process developed by de Nooy et al. in 1995, based on 2,2,6,6-tetramethylpiperidinyl-1-oxyl (TEMPO) radical-mediated oxidation with hypochlorite/bromide for the oxidation of the primary alcohol of the polysaccharides [105]. The as-assembled thiol-functionalized sponges had a honeycomb structure and were capable of selectively removing Hg^2+^ ions from an aqueous solution also containing other metal ions, with an adsorption capacity of up to 700 mg/g [97]. Interestingly, XPS (X-ray photoelectron spectroscopy) analysis revealed a key role of sulfur and oxygen in the adsorption of Hg^2+^, revealing the occurring interaction between sulfur/oxygen atoms and Hg^2+^. Thiol-modified cellulose sponges also demonstrated high reusability after three different adsorption–desorption cycles, using a 0.1 M aqueous solution of sodium edetate dihydrate to remove Hg^2+^.

The group of Zannagui et al. developed a new HEC-based adsorbent material using EDTA dianhydride (EDTAD) as a cross-linking agent [49]. In the study, a modified form of hydroxyethylcellulose was synthesized through the introduction of EDTA groups into the structure of the polysaccharide chain via a cross-linking reaction. EDTAD was used as the active agent, possessing two anhydride groups capable of easily reacting with the hydroxyl groups present in HEC. The evaluation of the adsorption capacity of Pb^2+^, Cu^2+^, Cd^2+^, and Zn^2+^ ions from aqueous solutions demonstrated that the modified HEC could be an efficient adsorbent for wastewater decontamination, with adsorption capacity values of 405.37, 265.47, 203.36, and 108.36 mg/g for Pb^2+^, Cu^2+^, Cd^2+^, and Zn^2+^, respectively. In this case, the selected cross-linking agent had a dual role, acting also as a functional moiety for improving the absorption capacity of the system.

### 3.3. Composite Hydrogels

The use of composite hydrogels has been explored as an effective strategy for improving the hydrogels’ ability to remove metal ions from water by increasing the number and the type of chemical functionalities involved in the adsorption process. For instance, the addition of cationic polymers confers the hydrogel the ability to capture oxygen anions such as chromate, thus expanding the range of applications in wastewater purification [106,107]. Furthermore, the possibility of tuning the composite hydrogel composition by modifying the ratio between components represents an additional benefit.

The introduction of vinyl monomers into cellulose-based hydrogels has been explored for imparting pH-sensitive properties, thus modulating the hydrogels’ ability to absorb heavy metal ions from water. Hemicellulose was modified via a transesterification reaction with glycidyl methacrylate (GMA) for the synthesis of vinyl macromonomers [108]. These systems succeeded in the removal of Cu^2+^, Cr^6+^, and As^5+^ ions. Specifically, the adsorption efficiency for the tested ions was studied as a function of the pH of the solution and the initial concentration of the metal ions in the aqueous solutions, revealing an adsorption capacity of 90 mg/g, 69 mg/g, and 60 mg/g for Cu^2+^, Cr^6+^, and As^5+^, respectively.

Hydroxyalkyl ether CMC has also found widespread use as a composite hydrogel in wastewater decontamination systems for removing heavy metal ions. The addition of polyacrylamide (PAM) increased the ability of the CMC-based hydrogels to capture heavy metals, such as Cu^2+^, Pb^2+^, and Cd^2+^, by 2.3, 3.6, and 1.5 times compared to the pristine CMC hydrogel, due to the presence of –C=O and –NH_2_ active in PAM [94].

Baiya et al. proposed the assembly of a CMC-based hydrogel (25%) mixed with PVA, employing glutaraldehyde as a cross-linking agent for the selective adsorption of Cu^2+^ ions [109]. Cellulose for CMC production was extracted from sugarcane bagasse, a waste product deriving from the crushing and pressing of sugar cane, with the aim of recycling waste materials in an eco-sustainable approach. To minimize the synthesis time and improve the reaction efficiency, the hydrogel was developed through a microwave-assisted irradiation process. The composite hydrogel revealed an adsorption capacity for Cu^2+^ at room temperature and pH 5.0 of 2.3 mg/g.

An excellent adsorbent material for the selective removal of Cr^6+^ ions was developed with the addition of the polyethylenimine (PEI) polymer to CMC, characterized by a notable quantity of imine groups [110]. The CMC/PEI composite hydrogel was produced with a simple one-step procedure, consisting of mixing an aqueous solution of CMC with an aqueous solution of PEI, to which the cross-linking agent epichlorohydrin was added. The protonated imine groups (at a slightly acidic pH) are able to adsorb Cr^6+^ ions through electrostatic interaction, while the oxidizing hydroxyl groups of CMC act by reducing Cr^6+^ ions to Cr^3+^. The in situ produced Cr^3+^ is immobilized through coordination with N and O and then removed through precipitation. The CMC/PEI composite hydrogel can be thus regenerated and reused, exhibiting a Cr^6+^ adsorption capacity higher than 2.0 mmol/g even after four cycles.

A composite hydrogel was assembled for the decontamination of aqueous matrices by removing cadmium and nickel based on CMC, microcrystalline cellulose (MCC), and xylan, using ethylene glycol diglycidyl ether as the cross-linking agent [111]. The reported maximum adsorption capacity was 61.44 for Cd^2+^ and 55.85 mg/g for Ni^2+^. The adsorption of metal ions can occur at the level of the carboxyl groups of the CMC, or even at the level of the hydroxyl groups of the MCC and xylan through ionic bonding.

**Figure 7:**
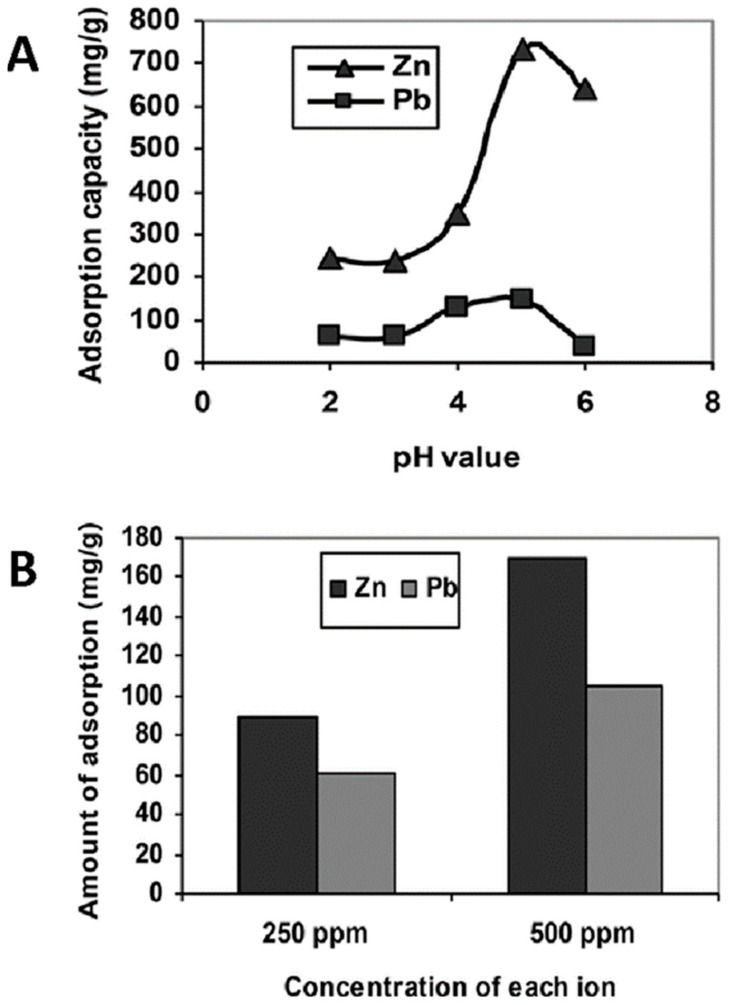
Influence of pH on heavy metal ion adsorption by CMC-graft-poly(acrylic acid)/montmorillonite hydrogel after 6 h of treatment (**A**). Competitive adsorption study at pH 5.0 of Pb^2+^ and Zn^2+^ from mixed solution with concentration of each ion fixed at 250 ppm or 500 ppm (**B**), from ref [112].

Batch adsorption tests of heavy metal ions from an aqueous solution were carried out by the group of Astrini et al., using a CMC-graft-poly(acrylic acid)/montmorillonite composite hydrogel as the adsorbent [112]. Clay minerals such as montmorillonite are introduced into the lattice structure of the polymer hydrogel in order to improve its mechanical properties. Their inclusion is motivated by their abundance, chemical and mechanical stability, and low cost, which makes them an attractive cellulose immobilization material. The impact of various parameters (such as pH and the presence of competing ions) on the adsorption capacity of the composite system was evaluated. The obtained results confirmed that the adsorption efficiency of metal ions by the composite hydrogel is significantly influenced by pH, with the maximum adsorption at pH 5.0 (Figure 7A). At this pH value, an adsorption capacity for the composite systems of 146.19 mg/g and 286.67 mg/g was obtained for Pb^2+^ and Zn^2+^ ions, respectively. Fourier transform infrared spectroscopy (FTIR) spectra before and after adsorption demonstrated that the complex formation between metal ions and the –COOH groups of the hydrogel is the main adsorption mechanism of heavy metal ions. Furthermore, competitive adsorption tests revealed that the adsorption capacity of the hydrogel for Zn^2+^ ions is higher than that for Pb^2+^ ions (Figure 7B). Desorption studies confirmed the effective metal ion desorption from the hydrogel, which could be reused twice through washing with a low pH solution.

**Figure 8:**
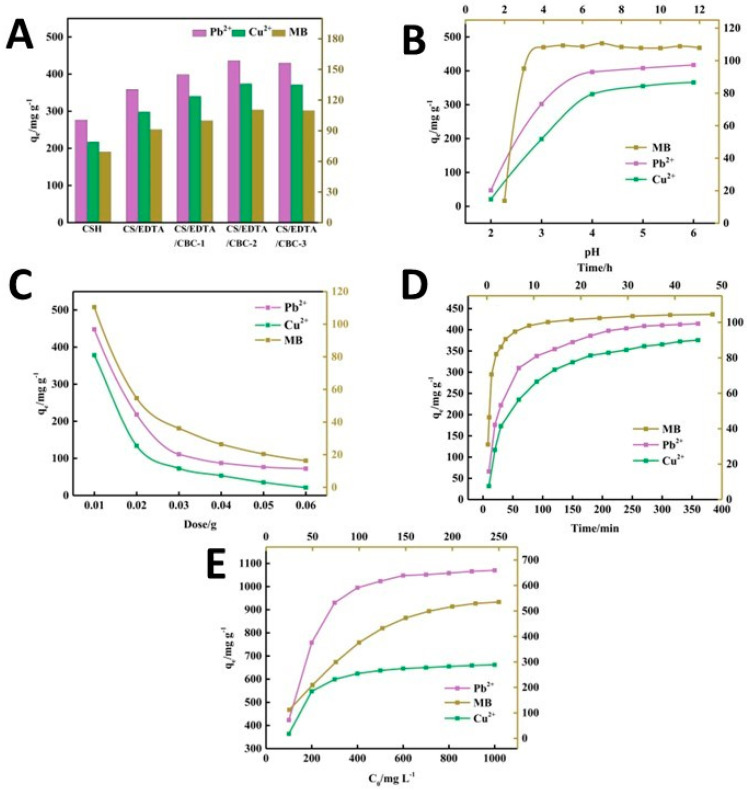
The impact of different parameters on the adsorption capacity: the composition of the adsorbent gel (**A**), pH (**B**), the amount of the adsorbent (**C**), the treatment time (**D**), and the initial concentration of the contaminant (**E**), from ref. [113]. CS: CH (chitosan); CBC: porous cotton biochar.

A novel acrylic acid/ethylenediaminetetraacetic acid (EDTA) biochar-based chitosan (CH) composite hydrogel was developed through radical polymerization from degreased waste cotton [113], mainly consisting of cellulose, hemicellulose, and lignin. The resulting hydrogel was able to adsorb Pb^2+^ and Cu^2+^ ions efficiently, with an adsorption capacity of 1105.78 and 678.04 mg/g, respectively. As shown in Figure 8, the impact of several factors on the adsorption capacity of the composite hydrogel was evaluated, finding that the recovery of Pb^2+^ and Cu^2+^ ions from the adsorbent was at an optimal level even after five cycles. The composition of the adsorbent plays a crucial role in the effectiveness of adsorption. As shown in Figure 8A, the adsorption performance of CH/EDTA was significantly improved compared to that of the CH hydrogel, due to the addition of EDTA which enhances the chelating effect of contaminants. In parallel, an increase in the amount of porous cotton biochar (CBC) increases the adsorption capacity of the hydrogel. This occurs due to the addition of numerous oxygen- and nitrogen-containing functional groups on the surface of the CBC after its acid–base modification, thus providing more anchoring sites for pollutants. However, once the CBC content reaches 0.03 g, the adsorption capacity stops increasing significantly, as a state of supersaturation of the CBC content is reached.

A new CH/cellulose composite sponge adsorbent system for the selective and rapid removal of Hg^2+^ ions from aqueous solutions was successfully developed through simple cross-linking with glutaraldehyde and a freeze-drying procedure [114]. The CH/cellulose composite system showed in the first two minutes an adsorption of more than 97% of the maximum adsorption capacity (equal to 495 mg/g with an initial concentration of 598 mg/L) in aqueous solutions. In addition, the composite system showed excellent selectivity for the adsorption of Hg^2+^ ions, with a selectivity coefficient 35, 277, and 461 times higher than that for Cd^2+^, Pb^2+^, and Cu^2+^ ions, respectively. The selectivity coefficient was determined using the separation factor, considering the distribution coefficients of Hg^2+^ ions and interfering metal ions. XPS and FTIR studies confirmed that the main mechanism of Hg^2+^ ion adsorption is a chelation reaction with N ligand and O-containing functional groups. In addition, washing with 1 M nitric acid allows the CH/cellulose composite system to be effectively regenerated, with slightly lower mechanical strength preserving an 85% adsorption capacity after five cycles.

**Figure 9:**
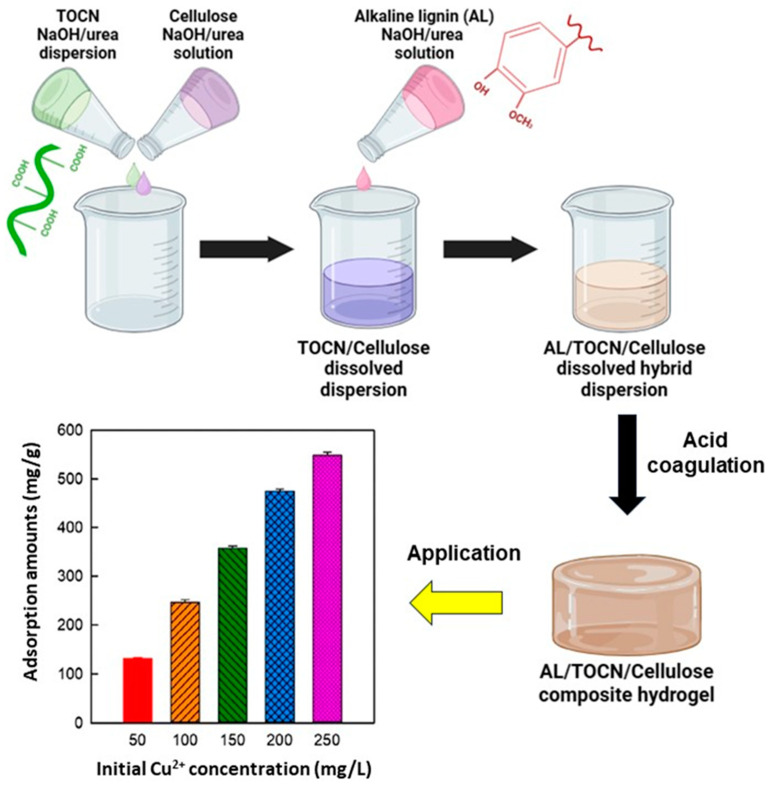
Synthesis schematic diagram and amount of Cu^2+^ adsorbed on the alkaline lignin/TOCN/dissolved cellulose composite hydrogel (10/10/80) at different initial Cu^2+^ ion concentrations, adapted from ref. [115].

Another lignocellulose-based composite hydrogel system was developed by dispersing TEMPO-oxidized cellulose nanofibrils (TOCNs) in an aqueous solution of 7% NaOH and 12% urea by weight at room temperature [115]. In the same system, dissolved cellulose was produced at subzero temperatures. The TOCN dispersion and cellulose solution were mixed at different temperatures based on their temperature-dependent solubility. The composite hydrogels were obtained by mixing a cellulose solution, TOCN dispersion, and alkaline lignin solution in an aqueous NaOH/urea solution. The resulting hydrogel showed an excellent adsorption capacity for Cu^2+^, equal to 540.7 mg/g (achieved after 45 min with an initial Cu^2+^ concentration of 250 mg/mL) (Figure 9). According to authors, the adsorption process of Cu^2+^ ions by composite hydrogels can be divided into two distinctive phases. Initially, Cu^2+^ ions diffuse onto the surface of the composite hydrogels from the surrounding solution. Subsequently, Cu^2+^ ions further bind to the active adsorption sites (phenolic and carboxyl groups from TOCNs and lignin) present on the composite hydrogels via complexation. In addition, the 3D porous network structure, which provides the maximum surface area and an abundant pore structure, leads to the exposure of many active sites for the complexation of Cu^2+^ ions.

TEMPO negatively charged oxidized cellulose nanofibers (TOCNFs) were used for the synthesis of a composite system through the self-assembly of nanocellulose and nanochitin [116]. Electrostatic forces guided the self-assembly between TOCNFs and the positively charged partially deacetylated chitin nanofibers at room temperature without the use of cross-linking agents. The resulting hybrid hydrogel was thus physically cross-linked through electrostatic interactions and H-bonding between TOCNFs and chitin nanofibers. Subsequent freeze-drying of the hybrid hydrogel resulted in an aerogel with a tightly interconnected porous structure and extraordinary adsorption capacity of As(III) ion (217 mg/g) at neutral pH.

TEMPO-oxidized nanocellulose was used also to produce a composite system with sodium alginate and carboxymethyl chitosan, using Ca^2+^ as a cross-linking agent [117]. Carboxymethyl chitosan is a derivative of chitosan characterized by a high number of free –OH, –COOH, and –NH_2_ groups, thus offering several binding sites for heavy metal ions. The gel produced was used in adsorption tests of Cu^2+^ and Pb^2+^ from aqueous solutions, showing an excellent adsorption efficiency for both ions, with an adsorption capacity of 169.94 mg/g for Cu^2+^ and 472.59 mg/g for Pb^2+^. In addition, the composite system maintained a high adsorption efficiency even after five adsorption–desorption cycles. Interestingly, FTIR analysis before and after the adsorption of Cu^2+^ and Pb^2+^ confirmed the involvement of –OH, –COOH, –NH_2_ groups, as well as methyl and methylene groups in the adsorption, demonstrating the key role of different components in determining the adsorption performances of the composite hydrogel.

A novel composite hydrogel based on microcrystalline cellulose (MCC) and CH was recently proposed, with the integration of polydopamine (PDA) and polyethyleneimine (PEI) for effective adsorption of ions from wastewater [118]. Thanks to the presence of several active adsorption sites, the MCC-PDA-PEI/CH-PDA-PEI composite system showed excellent adsorption capacities, equal to about 434.8, 277.7 and 261.8 mg/g for Cu^2+^, Zn^2+^, and Ni^2+^ ions, respectively, in a single ion experiment. In a multi-ion adsorption study, the composite hydrogel showed slight selectivity in the removal of Cu^2+^ ions. Furthermore, the results of FTIR studies suggested metal ion chelation as a possible adsorption mechanism. Repeated adsorption/desorption tests revealed that the hydrogel maintained an adsorption capacity of more than 40% after four cycles.

In a recent study, MCC was used for the synthesis of a biodegradable MCC–gelatin adsorbent for the adsorption of Cd^2+^, Pb^2+^, and Cr^3+^ ions from aqueous solutions [119]. The presence of several functional groups, such as –COOH groups, –OH groups, and amide groups (–CONH_2_), could facilitate the adsorption of heavy metal ions. The characterization of the composite hydrogel with FTIR analysis, scanning electron microscopy analysis (SEM), and energy dispersive X-ray analysis confirmed that the gelatin was intercalated within the microcrystalline cellulose matrix. Performing batch adsorption studies, the impact of several parameters including pH, adsorbent dosage, and initial metal ion concentration on the removal capacity of heavy metal ions was evaluated. Specifically, it was observed that there was a maximum adsorption efficiency for Cd^2+^, Pb^2+^, and Cr^3+^ of 95%, 88%, and 70%, respectively, at pH 6 with an initial concentration of each metal ion of 60 ppm and an adsorbent dosage of 1.0 g L^−1^.

Rodriguez et al. [120] developed free radical cross-linking composite chitosan-g-poly(acrylic acid) hydrogels filled with cellulose nanowhiskers (CNWs) and evaluated the absorption capacity for Pb^2+^ and Cu^2+^ ions from water. In the synthesis of the composite hydrogel by free radical cross-linking, three key components are involved such as N,N′-methylenebisacrylamide (MBA), which acts as a cross-linker, helping to stabilize the structure of the hydrogel, potassium persulfate (KPS), a polymerization initiator, and N,N,N′,N′-tetramethylethylenediamine (TEMD), which acts as an accelerator of the polymerization reaction. For these composite systems, the maximum adsorption for Pb^2+^ (818.4 mg/g) and Cu^2+^ (325.5 mg/g) was obtained within the first 30 min, at pH 4.0 and using 20 mg of the composite matrix with a CNW concentration of 10% (*w*/*w*). FTIR analysis revealed that functional groups (mainly –COOH and –OH) in composite hydrogels act as coordination sites for the metal ions. Desorption studies showed that the composite system can be regenerated (flushing with a 0.1 M HCl solution) and reused for up to five cycles without significant loss of efficiency.

Sphere-shaped composite systems have recently been proposed as possible adsorbents for the treatment of wastewater contaminated with heavy metal ions. In a recent work [121], a composite adsorbent based on cellulose nanofibers and sodium alginate was prepared through a simple cross-linking process for the efficient removal of Pb^2+^. The spheres were produced by mixing an aqueous solution of sodium alginate with an aqueous solution of cellulose nanofibers under stirring. A suspension of calcium phosphate (CaP) was then added and left stirring until a uniform dispersion was obtained, after which a CaCl_2_ solution was added. Overnight stirring resulted in the production of solidified cellulose nanofiber/sodium alginate/CaP beads. Subsequent stirring of the spheres in HCl (pH = 2.0) resulted in the disintegration of the CaP within the spheres, thus obtaining composite spheres with a porous structure, as revealed by scanning electron microscopy. XPS analysis confirmed the involvement of -OH and -COOH groups in the adsorption process involving electrostatic attraction and complexation. Also, the effect of temperature on the adsorption of Pb^2+^ ions was studied, revealing an increase in the limiting adsorption capacity as the treatment temperature increased, with a limiting adsorption capacity of 280.90 mg/g at 291 K, 307.69 mg/g at 297 K, and 318.47 mg/g at 303 K. Regeneration experiments with 1 M HCl showed that the adsorption capacity of Pb^2+^ on the beads remained satisfactory, with an adsorption above 80% after five cycles.

**Figure 10:**
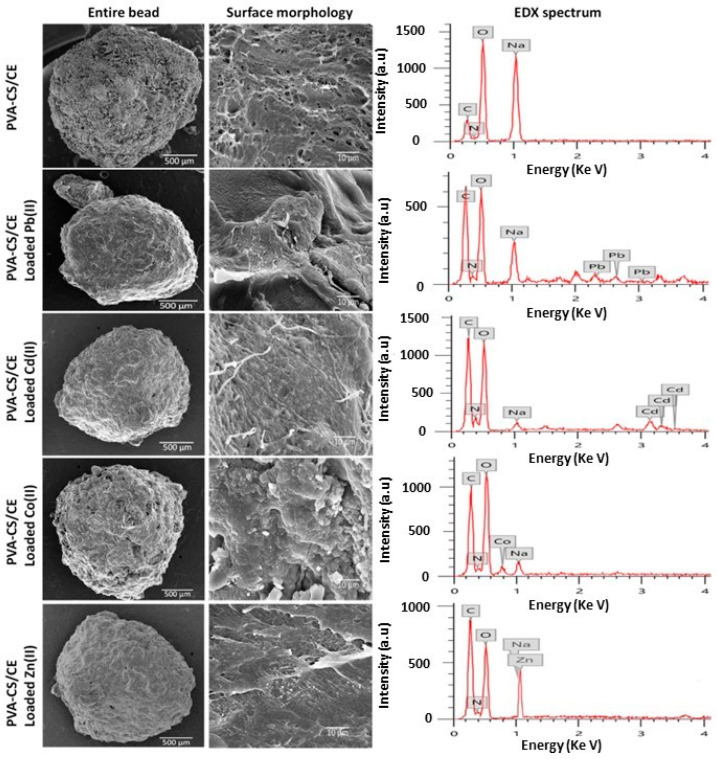
SEM images of surface of PVA/chitosan/cellulose hydrogel spheres before and after adsorption of heavy metal ions (**left**). EDX spectra of hydrogel spheres before and after adsorption of heavy metal ions (**right**), from ref. [122].

More recently, Aljar et al. [122] proposed composite beads made from PVA, chitosan, and cellulose for the removal of Pb^2+^, Cd^2+^, Zn^2+^, and Co^2+^ from water. The spheres were fabricated via a simple physical cross-linking method, in which an aqueous solution of PVA was mixed with an acidic aqueous solution of chitosan under continuous stirring at 70 °C, followed by cellulose powder addition. Subsequent dropwise addition of the obtained solution to a NaOH solution led to the formation of the hydrogel spheres. The morphology and major element composition of the composite spheres were analyzed before and after heavy metal ion adsorption via SEM and energy dispersive X-ray (EDX). SEM images revealed that the hydrogels possessed a spherical or slightly oval shape with a compact surface and a well cross-linked structure (Figure 10). In general, after adsorption, it was observed that the surface structure of the spheres remained as a well-cross-linked structure, even if the channels were more compact and the pores had a significantly smaller diameter. EDX spectra (Figure 10) clearly showed the characteristic peaks of heavy metal ions, confirming their adsorption. Adsorption studies also showed the efficient removal of Pb^2+^, Cd^2+^, Zn^2+^, and Co^2+^ ions, with 99, 95, 92, and 84% removal within 60 min. After regeneration of the spheres with 1 M HCl, the composite hydrogel maintained a high removal efficiency, which remained above 80% even after five adsorption–desorption cycles.

Table 3 reports the capacity adsorption values for the above presented cellulose-based hydrogels and composite adsorbents based on cellulose derivatives.

## 4. Conclusions

Polymer-based hydrogels, due to their ability to absorb large volumes of water, have emerged as a promising alternative material for the development of water decontamination processes. Herein, we have provided an overview of recent studies exploring the use of cellulose or cellulose derivative-based hydrogels for wastewater processing to remove heavy metal contaminants. Several properties have made cellulose and its derivatives an interesting class of materials for the remediation of contaminated water, including high biodegradability, abundance, low cost, optimal stability in a wide variety of conditions, and the large number of surface functional groups. Furthermore, since non-covalent interactions, such as hydrogen bonds and ion pairs, are commonly involved in the removal of heavy metal contaminants, cellulose-based systems can be potentially reused for several cycles.

Although they are the subject of numerous research studies and some pilot trials, their large-scale adoption in real water bodies is still far from actual implementation.

This is due to the possible effect of several additional factors when moving from laboratory-scale applications to the real environment, which can significantly affect metal ion adsorption processes. Along with metal ions, real wastewater contains a variety of contaminants, including microplastics, dyes, drugs, oils, detergents, pesticides, and fertilizers. Therefore, rather than merely concentrating on the investigation of a single metal ion, future adsorption research should be expanded not only to solutions containing different metal ions for evaluating possible cross-interference, but above all, to multipollutant evaluation.

Other physical/chemical characteristics of real water bodies, namely, pH, temperature, salinity, dissolved oxygen, and light exposure, can affect metal ion adsorption processes due to their natural variability which possibly represents an additional factor to be considered. Nonetheless, it is rare to find in the literature the testing of these hydrogels for real water samples or under simulated environmental conditions. The effectiveness of hydrogels under challenging environmental conditions must therefore be prioritized.

Environmental conditions can severely affect also cellulose-based hydrogel stability. As mentioned above, their biodegradability represents an additional value for their environmental applications as it means complete and safe degradation without toxic products being released. At the same time, their stability in real water naturally hosting different microorganisms must be considered and optimized to find a reasonable compromise between adsorption process kinetics and biodegradation timescales. Also in this sense, hydrogel tests in real environments are mandatory.

Cost evaluation represents a key aspect for promoting a technology transfer to the real world. In the case of hydrogels for environmental applications, costs are related not only to fabrication processes, but also to their regeneration capacity that determines their economic viability. However, although the hydrogel regeneration capacity is evaluated in a number of investigations, in most cases, the adsorption process is mostly focused on, with little or no attention given to effective hydrogel reuse. More in-depth studies should be conducted in this context to comprehend the regeneration capability of hydrogels during various cycles. Furthermore, the developed hydrogel regeneration processes must be sustainable not only in terms of costs but also in terms of environmental impact: safe post-treatment techniques for heavy metal desorption should be preferred for guaranteeing environment and operator protection during the whole process.

Importantly, the practical applicability of hydrogels in wastewater treatment can be eased by their integration with flowing beds, adsorption columns, and other devices that can be easily handled by simply exposing the system firstly to contaminated water and then to regeneration solutions. This further technological step should be considered in the design of hydrogels as it could be very favorable for their large-scale application, especially in the case of bead-shaped hydrogels. As discussed in the presented literature survey, such hydrogel format has been successfully applied to wastewater treatment studies, taking advantage of high surface area, which results in better contact with water and a larger adsorption area. Their packing within properly designed devices allows for their exposure to uniform waterflow and makes their handling easier, speeding up the whole process.

These aspects, if critically evaluated in studies focused on the development of cellulose-based adsorbents, can surely help bridge the gap between laboratory research and practical application, providing critical information for the success of cellulose-based hydrogels in removing metal ions in real environmental conditions.

## Figures and Tables

**Table 1:** The kinetic models used for heavy metal adsorption on adsorbents based on cellulose and its derivatives.

Model	Equation	Significance	Adsorbent	Reference
**Pseudo-First-Order**	**dq_t_/dt = k_1_ (q_e_ − q_t_)** (dq_t_/dt) represents the rate of change in the quantity of adsorbate with respect to time. (k_1_) is the pseudo-first-order rate constant. (q_e_) is the equilibrium adsorption capacity (the maximum amount of ions that can be adsorbed by the adsorbent). (q_t_) represents the amount of ions adsorbed at a specific time (t).	It is applicable in the initial phase before reaching the equilibrium absorption capacity.	*M. speciose* cellulose (MSCC)/chitosan cellulose fiber was extracted from pineapple leaves and modified with CM.	[92] [93]
**Pseudo-Second-Order**	**dq_t_/dt = k_2_ (q_e_ − q_t_)^2^** (dq_t_/dt) represents the rate of change in the quantity of adsorbate with respect to time. (k_2_) is the pseudo-second-order rate constant. (q_e_) is the equilibrium adsorption capacity (the maximum amount of ions that can be adsorbed by the adsorbent). (q_t_) represents the amount of ions adsorbed at a specific time (t).	Chemical adsorption represents the rate-limiting step, involving valence forces for chemical coordination through the sharing or exchange of electrons between the adsorbent and heavy metals (ion exchange). The occupancy rate of adsorption sites is proportional to the square of the number of unoccupied sites.	cellulose fiber was extracted from pineapple leaves and modified with EDTA. CMC/PAM CMC-based bead-like	[93] [94] [95]

**Table 2:** Main adsorption isotherm models for heavy metal ion adsorption by cellulose-based hydrogels and its derivatives.

Model	Equation	Significance	Adsorbent	Reference
**Langmuir**	**q_e_ = (q_max_ K_L_ C_e_)/1 + (K_L_ C_e_)** (q_e_): amount of adsorbed metal ions per unit mass of adsorbent at equilibrium. (C_e_): metal ion concentration in solution at equilibrium. (K_L_): Langmuir binding constant. (q_max_): maximum amount of metal adsorbed per unit weight of adsorbent.	Describes monolayer chemical adsorption on a homogeneous surface.	CMC Thiol-modified cellulose sponges.	[96] [97]
**Freundlich**	**q_e_ = K_F_ (C_e_)^1/n^** (q_e_): amount of adsorbed metal ions per unit mass of adsorbent at equilibrium. (C_e_): metal ion concentration in solution at equilibrium. (K_F_): Freundlich isotherm constant related to adsorption capacity. (n): constant related to adsorption intensity.	Describes multilayer physical adsorption on a non-uniform surface.	CMC-based bead-like	[95]
	**q_e_ = (RT/b_T_) ln(K_T_C_e_)**			
**Temkin**	(q_e_): amount of adsorbed metal ions per unit weignt of adsorbent at equilibrium. (C_e_): metal ion concentration in solution at equilibrium. (K_T_): Temkin isotherm equilibrium binding constant corresponding to the maximum binding energy. (b_T_): Temkin isotherm constant. (R): universal gas constant. (T): absolute temperature (°K).	Describes the indirect interaction between the adsorbent and the adsorbate and shows a linear decrease in heat of adsorption with increasing surface coverage of the adsorbent.	Cellulose grafted with the vinyl glycidyl methacrylate monomer.	[98]

**Table 3:** Composition and adsorption capacity of different cellulose-based hydrogels used for heavy metal absorption.

	Cellulose or Cellulose Derivative	Additional Polymer	Other Components or Modifications	Adsorption Capacity	Ref.
**Non-composite hydrogels**	CMC	―	epichlorohydrin as cross-linking agent	Cu^2+^: 6.49 mmol/g Pb^2+^: 5.15 mmol/g Ni^2+^: 4.06 mmol/g	[96]
	CMC	―	aluminum nitrate as cross-linking agent	Pb^2+^: 550 mg/g Ni^2+^: 620 mg/g Co^2+^: 760 mg/g	[95]
	Cellulose from bamboo fibers	―	titanate nanotubes	Cu^2+^: 176.4 mg/g Pb^2+^: 613.5 mg/g Sr^2+^: 113.3 mg/g Fe^2+^: 158.2 mg/g Cd^2+^: 294.6 mg/g Zn^2+^: 187.8 mg/g Cr^3+^: 97.8 mg/g	[99]
	Cellulose fiber from pineapple leaves	**―**	modification with carboxymethyl group (CM) and ethylenediaminetetraacetic acid (EDTA) group to produce Cell-CM and Cell-EDTA, respectively	**For Cell-CM** Pb^2+^: 63.4mg/g Cd^2+^: 23 mg/g **For Cell-EDTA** Pb^2+^: 41.2mg/g Cd^2+^: 33.2 mg/g	[93]
	HEC	―	EDTA dianhydride (EDTAD) as cross-linking agent	Pb^2+^: 405.37 mg/g Cu^2+^: 265.47 mg/g Cd^2+^: 203.36 mg/g Zn^2+^: 108.36 mg/g	[49]
	Ginger fibers	―	citric acid (C_6_H_8_O_7_) and hydrochloric acid (HCl)	Cu^2+^: 45.053 mg/g	[103]
	Jute	―	sodium nitrite and nitric acid	Pb^2+^: 2270 mg/g	[104]
**Composite** **hydrogels**	CMC and microcrystalline cellulose	Xilan	ethylene glycol diglycidyl ether as a crosslinking agent	Cd^2+^: 61.44 mg/g Ni^2+^: 55.85 mg/g	[111]
	CMC	Polyacrylic acid	montmorillonite		
				Pb^2+^: 146.19 mg/g Zn^2+^: 286.67 mg/g	[112]
	CMC	PVA	―	Ag^+^: 8.4 mg/g	[57]
	Degreasing cotton	Chitosan	ethylenediaminetetraacetic acid (EDTA)	Pb^2+^: 1105.78 mg/g Cu^2+^: 678.04 mg/g	[113]
	Cellulose	Chitosan	glutaraldehyde (EGDE) as crosslinking agent	Hg^2+^: 495 mg/g	[114]
	TEMPO-oxidized negatively charged (unidimensional) cellulose nanofibers (TOCNFs)	Partially deacetylated chitin nanofibers	―	As(III) ion: 217 mg/g	[116]
	TEMPO-oxidized nanocellulose	Carboxymethyl chitosan and sodium alginate	Ca^2+^ as cross-linking agent	Pb^2+^: 472.59 mg/g Cu^2+^: 169.94 mg/g	[117]
	Microcrystalline cellulose	Chitosan, polydopamine (PDA), and polyethyleneimine (PEI)	―	Cu^2+^: 434.8 mg/g Zn^2+^: 277.7 mg/g Ni^2+^ 261.8 mg/g	[118]
	Cellulose nanowhiskers (CNWs)	Chitosan-g-poly (acrylic acid)	MBA as cross-linking agent and TEMED as catalyst	Pb^2+^: 818.4 mg/g Cu^2+^: 325.5 mg/g	[120]

## Abbreviations

CMC	carboxy methyl cellulose
–COOH	carboxyl groups
CCNF	carboxylated cellulose nanofiber
CM	carboxymethyl group
CH	chitosan
CA	citric acid
EDTA	ethylenediaminetetraacetic acid group
EGDE	glutaraldehyde
HEC	hydroxyethyl cellulose
–OH	hydroxyl groups
MC	methylcellulose
MCC	microcrystalline cellulose
CNF	nanofibrillated cellulose
NOCNF	nitro-oxidized carboxycellulose nanofibers
PVA	poly(vinyl alcohol)
TOCNF	TEMPO-oxidized negatively charged (unidimensional) cellulose nanofiber
XRD	X-ray diffraction
TEMED	N,N,N′,N′-tetramethylethylenediamine
MBA	N,N′-methylenebisacrylamide

## References

[1] Soliman N.K., Moustafa A.F. (2020). Industrial solid waste for heavy metals adsorption features and challenges; A review. J. Mater. Res. Technol..

[2] Diaconu L.I., Covaliu-Mierlă C.I., Păunescu O., Covaliu L.D., Iovu H., Paraschiv G. (2023). Phytoremediation of Wastewater Containing Lead and Manganese Ions Using Algae. Biology.

[3] Ding C., Chen J., Zhu F., Chai L., Lin Z., Zhang K., Shi Y. (2022). Biological Toxicity of Heavy Metal (loid) s in Natural Environments: From Microbes to Humans. Front. Environ. Sci..

[4] Malitesta C., Di Masi S., Mazzotta E. (2017). From electrochemical biosensors to biomimetic sensors based on molecularly imprinted polymers in environmental determination of heavy metals. Front. Chem..

[5] Guascito M.R., Malitesta C., Mazzotta E., Turco A. (2008). Inhibitive determination of metal ions by an amperometric glucose oxidase biosensor: Study of the effect of hydrogen peroxide decomposition. Sens. Actuators B Chem..

[6] Guascito M.R., Malitesta C., Mazzotta E., Turco A. (2009). Screen-printed glucose oxidase-based biosensor for inhibitive detection of heavy metal ions in a flow injection system. Sens. Lett..

[7] Witkowska D., Słowik J., Chilicka K. (2021). Heavy metals and human health: Possible exposure pathways and the competition for protein binding sites. Molecules.

[8] Briffa J., Sinagra E., Blundell R. (2020). Heavy metal pollution in the environment and their toxicological effects on humans. Heliyon.

[9] Vinci G., Maddaloni L., Mancini L., Prencipe S.A., Ruggeri M., Tiradritti M. (2021). The Health of the Water Planet: Challenges and Opportunities in the Mediterranean Area. An Overview. Earth.

[10] Turco A., Monteduro A.G., Mazzotta E., Maruccio G., Malitesta C. (2018). An innovative porous nanocomposite material for the removal of phenolic compounds from aqueous solutions. Nanomaterials.

[11] Turco A., Pennetta A., Caroli A., Mazzotta E., Monteduro A.G., Primiceri E., de Benedetto G., Malitesta C. (2019). Easy fabrication of mussel inspired coated foam and its optimization for the facile removal of copper from aqueous solutions. J. Colloid Interface Sci..

[12] Turco A., Malitesta C., Barillaro G., Greco A., Maffezzoli A., Mazzotta E. (2015). A magnetic and highly reusable macroporous superhydrophobic/superoleophilic PDMS/MWNT nanocomposite for oil sorption from water. J. Mater. Chem. A.

[13] Turco A., Malitesta C. (2020). Removal of phenolic compounds from olive mill wastewater by a polydimethylsiloxane/oxMWCNTs porous nanocomposite. Water.

[14] López Y.C., Ortega G.A., Reguera E. (2022). Hazardous ions decontamination: From the element to the material. Chem. Eng. J. Adv..

[15] Obotey Ezugbe E., Rathilal S. (2020). Membrane technologies in wastewater treatment: A review. Membranes.

[16] Lech M., Gala O., Helińska K., Kołodzińska K., Konczak H., Mroczyński Ł., Siarka E. (2023). Membrane Separation in the Nickel-Contaminated Wastewater Treatment. Waste.

[17] Alkhadra M.A., Su X., Suss M.E., Tian H., Guyes E.N., Shocron A.N., Conforti K.M., de Souza J.P., Kim N., Tedesco M. (2022). Electrochemical methods for water purification, ion separations, and energy conversion. Chem. Rev..

[18] Qasem N.A., Mohammed R.H., Lawal D.U. (2021). Removal of heavy metal ions from wastewater: A comprehensive and critical review. NPJ Clean Water.

[19] Kurniawan S.B., Abdullah S.R.S., Imron M.F., Said N.S.M., Ismail N.I., Hasan H.A., Othman A.R., Purwanti I.F. (2020). Challenges and opportunities of biocoagulant/bioflocculant application for drinking water and wastewater treatment and its potential for sludge recovery. Int. J. Environ. Res. Public Health.

[20] Vishwakarma V. (2021). Recovery and recycle of wastewater contaminated with heavy metals using adsorbents incorporated from waste resources and nanomaterials-A review. Chemosphere.

[21] Gupta A., Sharma V., Sharma K., Kumar V., Choudhary S., Mankotia P., Kumar B., Mishra H., Moulick A., Ekielski A. (2021). A review of adsorbents for heavy metal decontamination: Growing approach to wastewater treatment. Materials.

[22] de Magalhães L.F., da Silva G.R., Peres A.E.C. (2022). Zeolite application in wastewater treatment. Adsorpt. Sci. Technol..

[23] Hassan M., Liu Y., Naidu R., Du J., Qi F., Donne S.W., Islam M.M. (2021). Mesoporous biopolymer architecture enhanced the adsorption and selectivity of aqueous heavy-metal ions. ACS Omega.

[24] Bashir S., Hina M., Iqbal J., Rajpar A.H., Mujtaba M.A., Alghamdi N.A., Wageh S., Ramesh K., Ramesh S. (2020). Fundamental concepts of hydrogels: Synthesis, properties, and their applications. Polymers.

[25] El Sayed M.M. (2023). Production of Polymer Hydrogel Composites and Their Applications. J. Polym. Environ..

[26] Mali P., Sherje A.P. (2022). Cellulose nanocrystals: Fundamentals and biomedical applications. Carbohydr. Polym..

[27] Mitura S., Sionkowska A., Jaiswal A. (2020). Biopolymers for hydrogels in cosmetics. J. Mater. Sci. Mater. Med..

[28] Magalhães M.I., Almeida A.P. (2023). Nature-Inspired Cellulose-Based Active Materials: From 2D to 4D. Appl. Biosci..

[29] Chen C., Xi Y., Weng Y. (2022). Recent advances in cellulose-based hydrogels for tissue engineering applications. Polymers.

[30] Topare N.S., Wadgaonkar V.S. (2023). A review on application of low-cost adsorbents for heavy metals removal from wastewater. Mater. Today Proc..

[31] Das S., Ghosh B., Sarkar K. (2022). Nanocellulose as sustainable biomaterials for drug delivery. Sens. Int..

[32] Badsha M.A., Khan M., Wu B., Kumar A., Lo I.M. (2021). Role of surface functional groups of hydrogels in metal adsorption: From performance to mechanism. J. Hazard. Mater..

[33] Cheng W., Zhu Y., Jiang G., Cao K., Zeng S., Chen W., Zhao D., Yu H. (2023). Sustainable cellulose and its derivatives for promising biomedical applications. Prog. Mater. Sci..

[34] Rongpipi S., Ye D., Gomez E.D., Gomez E.W. (2019). Progress and opportunities in the characterization of cellulose—An important regulator of cell wall growth and mechanics. Front. Plant Sci..

[35] Naomi R., Bt Hj Idrus R., Fauzi M.B. (2020). Plant-vs. Bacterial-derived cellulose for wound healing: A review. Int. J. Environ. Res. Public Health.

[36] Verma C., Mishra A., Chauhan S., Verma P., Srivastava V., Quraishi M.A., Ebenso E.E. (2019). Dissolution of cellulose in ionic liquids and their mixed cosolvents: A review. Sustain. Chem. Pharm..

[37] See W.Q., Jamaluddin J., Basar N., Bakar N.F.A., Azizan A., Alam M.N.H.Z., Lai J.C., Asmadi M., Adrus N. (2023). Green Chemistry Approaches to Cellulose Dissolution and Regeneration. Regenerated Cellulose and Composites: Morphology-Property Relationship.

[38] Morais E.S., Lopes A.M.D.C., Freire M.G., Freire C.S., Coutinho J.A., Silvestre A.J. (2020). Use of ionic liquids and deep eutectic solvents in polysaccharides dissolution and extraction processes towards sustainable biomass valorization. Molecules.

[39] Kayra N., Aytekin A.Ö. (2018). Synthesis of cellulose-based hydrogels: Preparation, formation, mixture, and modification. Cellulose-Based Superabsorbent Hydrogels.

[40] Aziz T., Farid A., Haq F., Kiran M., Ullah A., Zhang K., Li C., Ghazanfar S., Sun H., Ullah R. (2022). A review on the modification of cellulose and its applications. Polymers.

[41] Kabir S.F., Sikdar P.P., Haque B., Bhuiyan M.R., Ali A., Islam M.N. (2018). Cellulose-based hydrogel materials: Chemistry, properties and their prospective applications. Prog. Biomater..

[42] Kamide K. (2005). Characterization of molecular structure of cellulose derivatives. Cellulose and Cellulose Derivatives.

[43] McMullen R.L., Ozkan S., Gillece T. (2022). Physicochemical properties of cellulose ethers. Cosmetics.

[44] Nasution H., Harahap H., Dalimunthe N.F., Ginting M.H.S., Jaafar M., Tan O.O., Aruan H.K., Herfananda A.L. (2022). Hydrogel and effects of crosslinking agent on cellulose-based hydrogels: A review. Gels.

[45] Sabbagh F., Muhamad I.I., Pa’e N., Hashim Z. (2019). Strategies in improving properties of cellulose-based hydrogels for smart applications. Cellulose-Based Superabsorbent Hydrogels.

[46] Akter M., Bhattacharjee M., Dhar A.K., Rahman F.B.A., Haque S., Rashid T.U., Kabir S.F. (2021). Cellulose-based hydrogels for wastewater treatment: A concise review. Gels.

[47] Nasatto P.L., Pignon F., Silveira J.L., Duarte M.E.R., Noseda M.D., Rinaudo M. (2015). Methylcellulose, a cellulose derivative with original physical properties and extended applications. Polymers.

[48] Rahman M.S., Hasan M.S., Nitai A.S., Nam S., Karmakar A.K., Ahsan M.S., Shiddiky M.J.A., Ahmed M.B. (2021). Recent developments of carboxymethyl cellulose. Polymers.

[49] Zannagui C., Amhamdi H., El Barkany S., Jilal I., Sundman O., Salhi A., Chaouf S., Abou-Salama M., El Idrissi A., Zaghrioui M. (2020). Design, characterization and investigation of heavy metal ions removal by new cellulose-ether based adsorbent. Moroc. J. Chem..

[50] Durpekova S., Di Martino A., Dusankova M., Drohsler P., Sedlarik V. (2021). Biopolymer hydrogel based on acid whey and cellulose derivatives for enhancement water retention capacity of soil and slow release of fertilizers. Polymers.

[51] Mondal M.I.H., Haque M.O. (2019). Cellulosic hydrogels: A greener solution of sustainability. Cellulose-Based Superabsorbent Hydrogels.

[52] Morales A., Labidi J., Gullón P. (2020). Assessment of green approaches for the synthesis of physically crosslinked lignin hydrogels. J. Ind. Eng. Chem..

[53] Mah E., Ghosh R. (2013). Thermo-responsive hydrogels for stimuli-responsive membranes. Processes.

[54] Hu Y., Du Z., Deng X., Wang T., Yang Z., Zhou W., Wang C. (2016). Dual physically cross-linked hydrogels with high stretchability, toughness, and good self-recoverability. Macromolecules.

[55] Zhang H., Zhang F., Wu J. (2013). Physically crosslinked hydrogels from polysaccharides prepared by freeze–thaw technique. React. Funct. Polym..

[56] Bhaladhare S., Das D. (2022). Cellulose: A fascinating biopolymer for hydrogel synthesis. J. Mater. Chem. B.

[57] Wang L.Y., Wang M.J. (2016). Removal of heavy metal ions by poly (vinyl alcohol) and carboxymethyl cellulose composite hydrogels prepared by a freeze–thaw method. ACS Sustain. Chem. Eng..

[58] Guan Y., Bian J., Peng F., Zhang X.M., Sun R.C. (2014). High strength of hemicelluloses based hydrogels by freeze/thaw technique. Carbohydr. Polym..

[59] Ahmad Z., Salman S., Khan S.A., Amin A., Rahman Z.U., Al-Ghamdi Y.O., Akhtar K., Bakhsh E.M., Khan S.B. (2022). Versatility of hydrogels: From synthetic strategies, classification, and properties to biomedical applications. Gels.

[60] Zainal S.H., Mohd N.H., Suhaili N., Anuar F.H., Lazim A.M., Othaman R. (2021). Preparation of cellulose-based hydrogel: A review. J. Mater. Res. Technol..

[61] Zhang H., Wu X., Qin Z., Sun X., Zhang H., Yu Q., Yao M., He S., Dong X., Yao F. (2020). Dual physically cross-linked carboxymethyl cellulose-based hydrogel with high stretchability and toughness as sensitive strain sensors. Cellulose.

[62] Li Z., Lin Z. (2021). Recent advances in polysaccharide-based hydrogels for synthesis and applications. Aggregate.

[63] Han Y., Cao Y., Lei H. (2022). Dynamic Covalent Hydrogels: Strong yet Dynamic. Gels.

[64] Sapuła P., Bialik-Wąs K., Malarz K. (2023). Are Natural Compounds a Promising Alternative to Synthetic Cross-Linking Agents in the Preparation of Hydrogels?. Pharmaceutics.

[65] Zhao B., Jiang H., Lin Z., Xu S., Xie J., Zhang A. (2019). Preparation of acrylamide/acrylic acid cellulose hydrogels for the adsorption of heavy metal ions. Carbohydr. Polym..

[66] Coma V., Sebti I., Pardon P., Pichavant F.H., Deschamps A. (2003). Film properties from crosslinking of cellulosic derivatives with a polyfunctional carboxylic acid. Carbohydr. Polym..

[67] Wang C.C., Chen C.C. (2005). Physical properties of the crosslinked cellulose catalyzed with nanotitanium dioxide under UV irradiation and electronic field. Appl. Catal. A Gen..

[68] Demitri C., Del Sole R., Scalera F., Sannino A., Vasapollo G., Maffezzoli A., Ambrosio L., Nicolais L. (2008). Novel superabsorbent cellulose-based hydrogels crosslinked with citric acid. J. Appl. Polym. Sci..

[69] Raucci M.G., Alvarez-Perez M.A., Demitri C., Giugliano D., De Benedictis V., Sannino A., Ambrosio L. (2015). Effect of citric acid crosslinking cellulose-based hydrogels on osteogenic differentiation. J. Biomed. Mater. Res. Part A.

[70] Yan L., Shuai Q., Gong X., Gu Q., Yu H. (2009). Synthesis of microporous cationic hydrogel of hydroxypropyl cellulose (HPC) and its application on anionic dye removal. CLEAN Soil Air Water.

[71] Peng N., Wang Y., Ye Q., Liang L., An Y., Li Q., Chang C. (2016). Biocompatible cellulose-based superabsorbent hydrogels with antimicrobial activity. Carbohydr. Polym..

[72] Tanpichai S., Phoothong F., Boonmahitthisud A. (2022). Superabsorbent cellulose-based hydrogels cross-liked with borax. Sci. Rep..

[73] Jaishankar M., Tseten T., Anbalagan N., Mathew B.B., Beeregowda K.N. (2014). Toxicity, mechanism and health effects of some heavy metals. Interdiscip. Toxicol..

[74] Ali H., Khan E. (2018). What are heavy metals? Long-standing controversy over the scientific use of the term ‘heavy metals’—Proposal of a comprehensive definition. Toxicol. Environ. Chem..

[75] Balali-Mood M., Naseri K., Tahergorabi Z., Khazdair M.R., Sadeghi M. (2021). Toxic mechanisms of five heavy metals: Mercury, lead, chromium, cadmium, and arsenic. Front. Pharmacol..

[76] Kumar M., Seth A., Singh A.K., Rajput M.S., Sikandar M. (2021). Remediation strategies for heavy metals contaminated ecosystem: A review. Environ. Sustain. Indic..

[77] Qiao A., Cui M., Huang R., Ding G., Qi W., He Z., Klemes J.J., Su R. (2021). Advances in nanocellulose-based materials as adsorbents of heavy metals and dyes. Carbohydr. Polym..

[78] Darban Z., Shahabuddin S., Gaur R., Ahmad I., Sridewi N. (2022). Hydrogel-based adsorbent material for the effective removal of heavy metals from wastewater: A comprehensive review. Gels.

[79] Elgarahy A.M., Elwakeel K.Z., Mohammad S.H., Elshoubaky G.A. (2021). A critical review of biosorption of dyes, heavy metals and metalloids from wastewater as an efficient and green process. Clean. Eng. Technol..

[80] Nath P.C., Debnath S., Sharma M., Sridhar K., Nayak P.K., Inbaraj B.S. (2023). Recent advances in cellulose-based hydrogels: Food applications. Foods.

[81] Zhou D., Zhang L., Guo S. (2005). Mechanisms of lead biosorption on cellulose/chitin beads. Water Res..

[82] Song M., Wang J., He J., Kan D., Chen K., Lu J. (2023). Synthesis of Hydrogels and Their Progress in Environmental Remediation and Antimicrobial Application. Gels.

[83] Foudazi R., Zowada R., Manas-Zloczower I., Feke D.L. (2023). Porous Hydrogels: Present Challenges and Future Opportunities. Langmuir.

[84] Hossain L., Raghuwanshi V.S., Tanner J., Wu C.M., Kleinerman O., Cohen Y., Garnier G. (2020). Structure and swelling of cross-linked nanocellulose foams. J. Colloid Interface Sci..

[85] Karoyo A.H., Wilson L.D. (2021). A review on the design and hydration properties of natural polymer-based hydrogels. Materials.

[86] Mushtaq F., Raza Z.A., Batool S.R., Zahid M., Onder O.C., Rafique A., Nazeer M.A. (2022). Preparation, properties, and applications of gelatin-based hydrogels (GHs) in the environmental, technological, and biomedical sectors. Int. J. Biol. Macromol..

[87] Garg S., Garg A., Vishwavidyalaya R.D. (2016). Hydrogel: Classification, properties, preparation and technical features. Asian J. Biomater. Res..

[88] Zhao C., Liu G., Tan Q., Gao M., Chen G., Huang X., Xu X., Li L., Wang J., Zhang Y. (2023). Polysaccharide-based biopolymer hydrogels for heavy metal detection and adsorption. J. Adv. Res..

[89] Kaliaraj G.S., Shanmugam D.K., Dasan A., Mosas K.K.A. (2023). Hydrogels—A Promising Materials for 3D Printing Technology. Gels.

[90] Batys P., Kivistö S., Lalwani S.M., Lutkenhaus J.L., Sammalkorpi M. (2019). Comparing water-mediated hydrogen-bonding in different polyelectrolyte complexes. Soft Matter.

[91] Kopač T., Abrami M., Grassi M., Ručigaj A., Krajnc M. (2022). Polysaccharide-based hydrogels crosslink density equation: A rheological and LF-NMR study of polymer-polymer interactions. Carbohydr. Polym..

[92] Chen X., Huang Z., Luo S.Y., Zong M.H., Lou W.Y. (2021). Multi-functional magnetic hydrogels based on Millettia speciosa Champ residue cellulose and Chitosan: Highly efficient and reusable adsorbent for Congo red and Cu^2+^ removal. Chem. Eng. J..

[93] Daochalermwong A., Chanka N., Songsrirote K., Dittanet P., Niamnuy C., Seubsai A. (2020). Removal of heavy metal ions using modified celluloses prepared from pineapple leaf fiber. ACS Omega.

[94] Godiya C.B., Cheng X., Li D., Chen Z., Lu X. (2019). Carboxymethyl cellulose/polyacrylamide composite hydrogel for cascaded treatment/reuse of heavy metal ions in wastewater. J. Hazard. Mater..

[95] Li S.S., Song Y.L., Yang H.R., An Q.D., Xiao Z.Y., Zhai S.R. (2020). Carboxymethyl cellulose-based cryogels for efficient heavy metal capture: Aluminum-mediated assembly process and sorption mechanism. Int. J. Biol. Macromol..

[96] Yang S., Fu S., Liu H., Zhou Y., Li X. (2011). Hydrogel beads based on carboxymethyl cellulose for removal heavy metal ions. J. Appl. Polym. Sci..

[97] Rong L., Zhu Z., Wang B., Mao Z., Xu H., Zhang L., Zhong Y., Sui X. (2018). Facile fabrication of thiol-modified cellulose sponges for adsorption of Hg^2+^ from aqueous solutions. Cellulose.

[98] Zhou Y., Hu X., Zhang M., Zhuo X., Niu J. (2013). Preparation and characterization of modified cellulose for adsorption of Cd(II), Hg(II), and acid fuchsin from aqueous solutions. Ind. Eng. Chem. Res..

[99] Xiong Y., Xu L., Jin C., Sun Q. (2020). Cellulose hydrogel functionalized titanate microspheres with self-cleaning for efficient purification of heavy metals in oily wastewater. Cellulose.

[100] Peng H., Fan G., Zheng X., Luo J., Zhou J. (2018). Impact of structure and morphology of titanate nanomaterials on Pb^2+^ adsorption in aqueous solution. Desalination Water Treat..

[101] Hara K., Iida M., Yano K., Nishida T. (2004). Metal ion absorption of carboxymethylcellulose gel formed by γ-ray irradiation: For the environmental purification. Colloids Surf. B Biointerfaces.

[102] Kamel S., Hassan E.M., El-Sakhawy M. (2006). Preparation and application of acrylonitrile-grafted cyanoethyl cellulose for the removal of copper (II) ions. J. Appl. Polym. Sci..

[103] Wang D., Yu H., Fan X., Gu J., Ye S., Yao J., Ni Q. (2018). High aspect ratio carboxylated cellulose nanofibers cross-linked to robust aerogels for superabsorption–flocculants: Paving way from nanoscale to macroscale. ACS Appl. Mater. Interfaces.

[104] Sharma P.R., Chattopadhyay A., Zhan C., Sharma S.K., Geng L., Hsiao B.S. (2018). Lead removal from water using carboxycellulose nanofibers prepared by nitro-oxidation method. Cellulose.

[105] De Nooy A.E., Besemer A.C., van Bekkum H. (1995). Highly selective nitroxyl radical-mediated oxidation of primary alcohol groups in water-soluble glucans. Carbohydr. Res..

[106] Li L., Guo J., Kang C., Song H. (2023). Reinforcement of Nanocomposite Hydrogel with Dialdehyde Cellulose Nanofibrils via Physical and Double Network Crosslinking Synergies. Polymers.

[107] Nicu R., Ciolacu F., Ciolacu D.E. (2021). Advanced functional materials based on nanocellulose for pharmaceutical/medical applications. Pharmaceutics.

[108] Sánchez J., Dax D., Tapiero Y., Xu C., Willför S. (2021). Bio-based hydrogels with ion exchange properties applied to remove Cu(II), Cr(VI), and As(V) ions from water. Front. Bioeng. Biotechnol..

[109] Baiya C., Nannuan L., Tassanapukdee Y., Chailapakul O., Songsrirote K. (2019). The synthesis of carboxymethyl cellulose-based hydrogel from sugarcane bagasse using microwave-assisted irradiation for selective adsorption of copper(II) ions. Environ. Prog. Sustain. Energy.

[110] Song L., Liu F., Zhu C., Li A. (2019). Facile one-step fabrication of carboxymethyl cellulose based hydrogel for highly efficient removal of Cr(VI) under mild acidic condition. Chem. Eng. J..

[111] Kundu D., Mondal S.K., Banerjee T. (2019). Development of β-cyclodextrin-cellulose/hemicellulose-based hydrogels for the removal of Cd(II) and Ni(II): Synthesis, kinetics, and adsorption aspects. J. Chem. Eng. Data.

[112] Astrini N., Anah L., Haryadi H.R. (2015). Adsorption of heavy metal ion from aqueous solution by using cellulose based hydrogel composite. Macromolecular Symposia.

[113] Fan X., Wang X., Cai Y., Xie H., Han S., Hao C. (2022). Functionalized cotton charcoal/chitosan biomass-based hydrogel for capturing Pb^2+^, Cu^2+^ and MB. J. Hazard. Mater..

[114] Zhang D., Wang L., Zeng H., Yan P., Nie J., Sharma V.K., Wang C. (2019). A three-dimensional macroporous network structured chitosan/cellulose biocomposite sponge for rapid and selective removal of mercury(II) ions from aqueous solution. Chem. Eng. J..

[115] Zhang L., Lu H., Yu J., Fan Y., Yang Y., Ma J., Wang Z. (2018). Synthesis of lignocellulose-based composite hydrogel as a novel biosorbent for Cu^2+^ removal. Cellulose.

[116] Zhang X., Elsayed I., Navarathna C., Schueneman G.T., Hassan E.B. (2019). Biohybrid hydrogel and aerogel from self-assembled nanocellulose and nanochitin as a high-efficiency adsorbent for water purification. ACS Appl. Mater. Interfaces.

[117] Li W., Zhang L., Hu D., Yang R., Zhang J., Guan Y., Lv F., Gao H. (2021). A mesoporous nanocellulose/sodium alginate/carboxymethyl-chitosan gel beads for efficient adsorption of Cu^2+^ and Pb^2+^. Int. J. Biol. Macromol..

[118] Godiya C.B., Revadekar C., Kim J., Park B.J. (2022). Amine-bilayer-functionalized cellulose-chitosan composite hydrogel for the efficient uptake of hazardous metal cations and catalysis in polluted water. J. Hazard. Mater..

[119] Sultana M., Ahmmed M.R., Ul Hoque M.I., Ebna Mohsen T., Mondol A., Rahaman M.H., Yesmin M., Rahman M.S., Nur Alam S.M. (2023). Biodegradable Composite of Gelatin Blend Microcrystalline Cellulose for Cd^2+^, Pb^2+^, and Cr^3+^ Adsorption from an Aqueous Solution. Adv. Polym. Technol..

[120] Rodrigues F.H., de C. (2019). Magalhães, C.E.; Medina, A.L.; Fajardo, A.R. Hydrogel composites containing nanocellulose as adsorbents for aqueous removal of heavy metals: Design, optimization, and application. Cellulose.

[121] Zhao H., Ouyang X.K., Yang L.Y. (2021). Adsorption of lead ions from aqueous solutions by porous cellulose nanofiber–sodium alginate hydrogel beads. J. Mol. Liq..

[122] Aljar M.A.A., Rashdan S., Almutawah A., El-Fattah A.A. (2023). Synthesis and Characterization of Biodegradable Poly (vinyl alcohol)-Chitosan/Cellulose Hydrogel Beads for Efficient Removal of Pb(II), Cd(II), Zn(II), and Co(II) from Water. Gels.

